# Machine Learning Leveraging Genomes from Metagenomes Identifies Influential Antibiotic Resistance Genes in the Infant Gut Microbiome

**DOI:** 10.1128/mSystems.00123-17

**Published:** 2018-01-09

**Authors:** Sumayah F. Rahman, Matthew R. Olm, Michael J. Morowitz, Jillian F. Banfield

**Affiliations:** a Department of Plant and Microbial Biology, University of California, Berkeley, Berkeley, California, USA; b Department of Surgery, University of Pittsburgh School of Medicine, Pittsburgh, Pennsylvania, USA; c Department of Earth and Planetary Sciences and Environmental Science, Policy and Management, University of California, Berkeley, Berkeley, California, USA; University of Trento

**Keywords:** *Clostridium difficile*, antibiotic resistance, genome-resolved metagenomics, infant, machine learning, microbiome, resistome

## Abstract

Antibiotic resistance in pathogens is extensively studied, and yet little is known about how antibiotic resistance genes of typical gut bacteria influence microbiome dynamics. Here, we leveraged genomes from metagenomes to investigate how genes of the premature infant gut resistome correspond to the ability of bacteria to survive under certain environmental and clinical conditions. We found that formula feeding impacts the resistome. Random forest models corroborated by statistical tests revealed that the gut resistome of formula-fed infants is enriched in class D beta-lactamase genes. Interestingly, *Clostridium difficile* strains harboring this gene are at higher abundance in formula-fed infants than *C. difficile* strains lacking this gene. Organisms with genes for major facilitator superfamily drug efflux pumps have higher replication rates under all conditions, even in the absence of antibiotic therapy. Using a machine learning approach, we identified genes that are predictive of an organism’s direction of change in relative abundance after administration of vancomycin and cephalosporin antibiotics. The most accurate results were obtained by reducing annotated genomic data to five principal components classified by boosted decision trees. Among the genes involved in predicting whether an organism increased in relative abundance after treatment are those that encode subclass B2 beta-lactamases and transcriptional regulators of vancomycin resistance. This demonstrates that machine learning applied to genome-resolved metagenomics data can identify key genes for survival after antibiotics treatment and predict how organisms in the gut microbiome will respond to antibiotic administration.

**IMPORTANCE** The process of reconstructing genomes from environmental sequence data (genome-resolved metagenomics) allows unique insight into microbial systems. We apply this technique to investigate how the antibiotic resistance genes of bacteria affect their ability to flourish in the gut under various conditions. Our analysis reveals that strain-level selection in formula-fed infants drives enrichment of beta-lactamase genes in the gut resistome. Using genomes from metagenomes, we built a machine learning model to predict how organisms in the gut microbial community respond to perturbation by antibiotics. This may eventually have clinical applications.

## Abstract (precis)

The process of reconstructing genomes from environmental sequence data (genome-resolved metagenomics) allows unique insight into microbial systems. We apply this technique to investigate how the antibiotic resistance genes of bacteria affect their ability to flourish in the gut under various conditions. Our analysis reveals that strain-level selection in formula-fed infants drives enrichment of beta-lactamase genes in the gut resistome. Using genomes from metagenomes, we built a machine learning model to predict how organisms in the gut microbial community respond to perturbation by antibiotics. This may eventually have clinical applications.

## INTRODUCTION

Antibiotic use steadily increased over the past several decades and is correlated with the prevalence of antibiotic resistance in bacteria (1). Widespread antibiotic resistance, in combination with the decline in development of new antibiotics, presents a major threat to human health (2). The gut microbiome is a reservoir for antibiotic resistance genes (3) and may be involved in the spread of resistance genes to pathogens (4–6). Additionally, antibiotics are often prescribed to treat infections without considering how the drug will affect the gut microbial community, which can lead to negative consequences for the human host (7). It is therefore important to study how the antibiotic resistance genes harbored by organisms in the gut microbiome impact community dynamics.

The preterm infant gut resistome is considered a research priority because premature infants are almost universally administered antibiotics during the first week of life (8). Early life is a critically important time for community establishment (9), and neonatal antibiotic therapies have both transient and persistent effects on the gut microbial community. Included among the many ways in which antibiotics have been shown to affect the microbiome are lower bacterial diversity (10), enrichment of *Enterobacteriaceae* (10, 11), reduction of *Bifidobacterium* spp. (12), and enrichment of antibiotic resistance genes (13), including those that have no known activity against the particular antibiotic administered (14). Previous studies have shown that the community composition of the infant microbiome is affected by diet, with artificial formula selecting for *Escherichia coli* and *Clostridium difficile* (15) and breast milk selecting for certain strains of *Bifidobacterium* (16). Hypotheses regarding the effect of birth mode on the microbiome are contested, with most studies finding that it has an effect on the gut microbiome (17–19) whereas some show no effect (20, 21). Gender (22) and maternal antibiotics administered before or during birth (23–25) also influence microbiome assembly.

Here we used genome-resolved metagenomics coupled with statistical and machine learning approaches to investigate the gut resistome of 107 longitudinally sampled premature infants. We show that certain antibiotic resistance genes in particular genomes affect how clinical factors influence the gut microbiome and, in turn, how the antibiotic resistance capabilities of a gut organism influence its growth and relative abundance.

## RESULTS AND DISCUSSION

### Antibiotic resistance of the premature infant microbiome.

A total of 107 premature infants were studied during the first 3 months of life. The median birth weight was 1,228 g (interquartile range [IQR] = 902 to 1,462), with 35% of the infants having extremely low (<1,000 g) birth weight and 65% of infants having birth weight of >1,000 g (see Table S1 and Text S1 in the supplemental material). Birth weight is closely linked to gestational age, which is divided into the following categories: late preterm (34-week to <37-week gestation), moderate preterm (32-week to <34-week gestation), very preterm (28-week to <32-week gestation), and extremely preterm (<28 weeks gestation) (26). Among the infants in this study, 30% were extremely preterm; such infants tend to have significant health problems, including higher rates of necrotizing enterocolitis and extreme dysbiosis of the microbiota (27). The majority (60%) of the infants in our study were classified as very preterm, just 10% of our infants were classified as moderate preterm, and no infants were classified as late preterm (Table S1 and Text S1). Because the infants in this study were mostly very or extremely preterm, it should be noted that the biological characteristics reported here are highly divergent from those of typical full-term infants (28).

10.1128/mSystems.00123-17.1 TEXT S1  Jupyter notebook with the code used to perform the computational and statistical analyses, along with the resulting plots. Download TEXT S1, PDF file, 0.4 MB. Copyright © 2018 Rahman et al. 2018 Rahman et al. This content is distributed under the terms of the Creative Commons Attribution 4.0 International license.

10.1128/mSystems.00123-17.2 TABLE S1  The metadata associated with each of the 107 infants in this study. Download TABLE S1, XLS file, 0.1 MB. Copyright © 2018 Rahman et al. 2018 Rahman et al. This content is distributed under the terms of the Creative Commons Attribution 4.0 International license.

**FIG 1:**
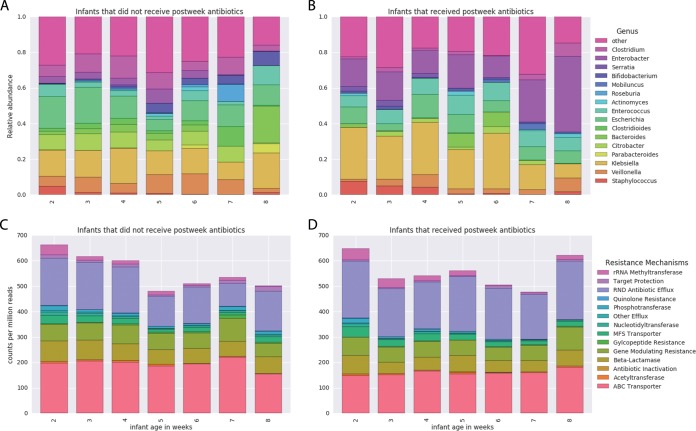
Microbiome and resistome of the premature infant gut microbial community. The numbers of samples included in each week’s average are as follows: for the infants that did not receive antibiotics after the first week, week 2 *n* = 197, week 3 *n* = 188, week 4 *n* = 110, week 5 *n* = 16, week 6 *n* = 20, week 7 *n* = 7, and week 8 *n* = 8; for the infants that received antibiotics after the first week, week 2 *n* = 72, week 3 *n* = 73, week 4 *n* = 53, week 5 *n* = 24, week 6 *n* = 16, week 7 *n* = 8, and week 8 *n* = 13. (A) The genus-level taxonomic composition of the gut community for the infants that did not receive antibiotics after the first week of life. (B) The genus-level taxonomic composition of the gut community for the infants that received antibiotics beyond the first week of life. (C) For the infants that do not receive antibiotics after the first week, the total resistance content of the premature infant gut microbiome has a slight negative correlation with age (*P* = 0.003.). (D) The resistance gene levels of infant microbiomes that were exposed to additional antibiotics did not display a significant trend (*P* = 0.265).

Longitudinal sampling of each infant resulted in a total of 902 samples that were sequenced and analyzed. All 107 infants received gentamicin and ampicillin during the first week of life, and 36 of those infants received additional antibiotics in the later weeks due to disease (Table 1). Data on the types of antibiotics given to the infants, along with the day of life (DOL) on which they were administered, are available in Table S2. All samples were subjected to Illumina short-read shotgun sequencing, and the sequence data were assembled using idba-ud (see Materials and Methods for details). Binning resulted in a dereplicated set of 1,483 genomes (Table S3). The taxonomic composition of these samples is typical for the premature infant gut (Fig. 1A and B). Resfams (29) annotations of predicted amino acid sequences from the resulting scaffolds (Table S4) revealed that the most abundant resistance mechanisms were resistance-nodulation-cell division (RND) efflux pumps and ATP-binding-cassette (ABC) transporters (Fig. 1C and D). Note that, in addition to their ability to contribute to antibiotic resistance, efflux pumps and transporters have been associated with stress response (30–32) and may reflect a rapidly changing environment during the first few months of life.

10.1128/mSystems.00123-17.3 TABLE S2  The metadata associated with each of the 902 samples sequenced in this study. Download TABLE S2, XLS file, 0.1 MB. Copyright © 2018 Rahman et al. 2018 Rahman et al. This content is distributed under the terms of the Creative Commons Attribution 4.0 International license.

10.1128/mSystems.00123-17.4 TABLE S3  Quality metrics and statistics of the Concoct genome bins. Download TABLE S3, XLS file, 0.4 MB. Copyright © 2018 Rahman et al. 2018 Rahman et al. This content is distributed under the terms of the Creative Commons Attribution 4.0 International license.

10.1128/mSystems.00123-17.5 TABLE S4  Annotations resulting from HMMER *hmmscan* search against the Resfams database using gathering threshold as cutoff values. Download TABLE S4, XLS file, 19 MB. Copyright © 2018 Rahman et al. 2018 Rahman et al. This content is distributed under the terms of the Creative Commons Attribution 4.0 International license.

**TABLE 1:** Infant characteristics

Characteristic	Value for infants who:	
	Received no antibiotics after the first week	Received antibiotics after the first week^a^
No. of samples	604	298
Total no. of infants^b^	71	36
No. of infants who received breast milk	52	32
No. of infants who were delivered by C-section	54	22
No. of infants of male sex	34	17
No. of infants with maternal antibiotics	24	20

^a^ The infants represented in the column corresponding to those who received antibiotics after the first week (right) were administered antibiotics while in the NICU beyond the first week of life due to late-onset sepsis, necrotizing enterocolitis, or another disease.

^b^ All 107 premature infants were in the neonatal intensive care unit (NICU) of the Magee-Women’s Hospital in Pittsburgh, PA.

For the infants that did not receive additional antibiotics (Fig. 1C), a decreasing trend in total antibiotic resistance potential was observed over time (*P* < 0.005) (Text S1). During the first week of life, empirical antibiotic therapy perturbs the microbiome by preferentially enriching for antibiotic-resistant organisms. This is consistent with prior results showing temporarily elevated resistance gene levels after administration of antibiotics (17). Microbial community recovery begins following this period. For the infants that received antibiotics after the first week of life (Fig. 1D), there was no consistent trend of decreasing resistance potential (Text S1). This suggests that administration of antibiotics to premature infants after the first week of life can prolong the enrichment of the resistome.

Approximately 20% of the resistance genes annotated by Resfams (Table S4) were not assignable to specific organisms in the microbiome (Table S5 and Text S1). This was partly due to some genes being carried on plasmids, which were excluded from the genomic analysis.

10.1128/mSystems.00123-17.6 TABLE S5  Genome resistance gene profiles display the count for each Resfams (resistance gene family) that appeared in each genome bin. Download TABLE S5, XLS file, 0.9 MB. Copyright © 2018 Rahman et al. 2018 Rahman et al. This content is distributed under the terms of the Creative Commons Attribution 4.0 International license.

### Formula feeding influences the gut resistome through strain-level selection.

Permutational multivariate analysis of variance (PERMANOVA) tests, which discern and isolate the effects of factors through partitioning of variance (33), were performed to investigate the effect of feeding regimen, delivery mode, gender, maternal antibiotics, and the infant’s current antibiotic therapy on the resistome (Text S1). Tests were performed on the resistomes of samples taken at weeks 2, 4, and 6 to avoid the bias of repeated measures in longitudinal sampling. At week 2, the distribution of antibiotic resistance genes seen with formula-fed infants was not significantly different from that seen with infants that received breast milk. However, a difference was detected at weeks 4 and 6 (*P* < 0.05), accompanied by an increase in effect size as assessed by PERMANOVA F-statistic (Table S6). This signals divergence of the resistomes of formula-fed and breast-fed infants over time. The PERMANOVA tests were not sensitive enough to detect any effects on the resistome resulting from delivery mode, gender, or antibiotics, which may have been because the test displays conservatism when variances are positively related to group sample size (34). Because these factors have been shown to alter the microbiota (19, 22, 24, 25), it is unlikely that the resistome was truly unchanged. Since feeding type was the only factor that produced a detectable response, we further investigated its effects.

10.1128/mSystems.00123-17.7 TABLE S6  Result tables of marginal PERMANOVAs with 9,999 random permutations for weeks 2, 4, and 6 performed on the antibiotic resistance gene content of infant samples as annotated by Resfams. The Bray-Curtis distance metric was used in the PERMANOVA, and Bonferroni corrections were applied to the *P* values to correct for multiple testing. Download TABLE S6, DOCX file, 0.1 MB. Copyright © 2018 Rahman et al. 2018 Rahman et al. This content is distributed under the terms of the Creative Commons Attribution 4.0 International license.

**FIG 2:**
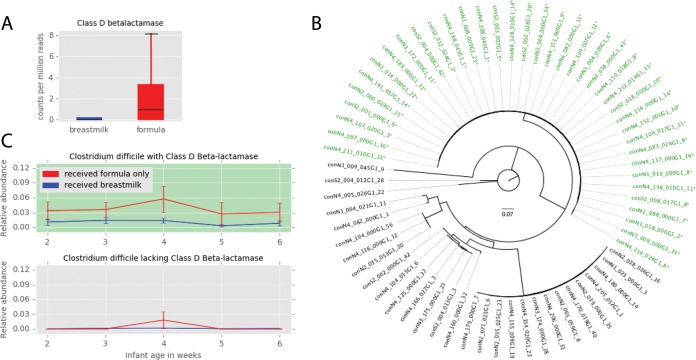
Formula feeding affects the resistome. (A) Class D beta-lactamase is enriched in formula-fed infants at 4 weeks of age (Mann-Whitney *U* = 66, Bonferroni-corrected *P* = 0.031). (B) Phylogenetic tree of *Clostridium difficile* genomes based on the ribosomal protein S3 gene. Names of genomes harboring a class D beta-lactamase are colored green and labeled with an asterisk. (C) The relative abundances of *C. difficile* genomes with class D beta-lactamase in formula-fed and breast-fed infants (top) (*n* = 38) and the relative abundances of *C. difficile* genomes lacking class-D betalactamase in formula-fed and breast-fed infants (bottom) (*n* = 29). Only the infants harboring *C. difficile* were included in calculations of average relative abundances.

Random forest models were used to classify resistomes as belonging to either a formula-fed baby or a breast-fed baby, and we used the feature importance scores of the trained models to select resistance genes for further study (Table 2). One of the four selected resistance genes was significantly associated with a feeding group: class D beta-lactamase was enriched in formula-fed infants (*P* < 0.05) (Fig. 2A). Genome-resolved analysis (Table S5 and S7) revealed that class D beta-lactamase genes are most frequently carried by *Clostridium difficile* (Text S1). Among the 67 *C. difficile* genomes in the dereplicated data set, 38 harbor a class D beta-lactamase gene. Phylogenetic analysis reveals that these 38 organisms are very closely related (Fig. 2B). To ascertain if this *C. difficile* strain is involved in the enrichment of class D beta-lactamase in the formula-fed infant gut resistome, the relative abundances of *C. difficile* with and without a class D beta-lactamase gene in the gut microbiome of breast-fed and formula-fed infants were assessed. *C. difficile* with a class D beta-lactamase gene was consistently more abundant than *C. difficile* lacking this gene among the infants that received only formula, while both types of *C. difficile* were low in relative abundance among the infants that received breast milk (Fig. 2C). Even with the lower relative abundance of some *C. difficile* strains, there was no significant difference in genome completeness and N50 between the two groups (Table S3 and Text S1), assuring us that there was no methodological issue that reduced our ability to detect beta-lactamase. Prior studies have reported an increased abundance of *C. difficile* in the gut microbiomes of formula-fed infants (15), but here we reveal that formula feeding enriches for a particular *C. difficile* strain.

10.1128/mSystems.00123-17.8 TABLE S7  The taxonomic assignment of each genome bin. Download TABLE S7, XLS file, 0.1 MB. Copyright © 2018 Rahman et al. 2018 Rahman et al. This content is distributed under the terms of the Creative Commons Attribution 4.0 International license.

**TABLE 2:** Features selected using the random forest Gini importance metric after training on resistomes of formula-fed infants and breast-fed infants

Resfams category	Feature importance score	Mann-Whitney U value	*P* value	Corrected *P* value^a^
ANT6	0.071	17	0.327	1
Class D beta-lactamase	0.089	66	0.008	0.031
*mexX*	0.098	11	0.106	0.426
*soxR* mutant	0.071	10	0.081	0.324

^a^ Bonferroni corrections were applied to the *P* values obtained from Mann-Whitney *U* tests to adjust for multiple testing.

Class D beta-lactamase hydrolyzes beta-lactam antibiotics (35), and there is no known connection between host diet and its antibiotic resistance function. It is thus unlikely that class D beta-lactamase offers a selective advantage to organisms in the gut of formula-fed infants, but this gene may be linked to other genes that confer an advantage. Pairwise correlations of the Resfams and KEGG metabolism modules in *C. difficile* genomes revealed that one KEGG module, the cytidine 5′-monophospho-3-deoxy-d-*manno*-2-octulosonic acid (CMP-KDO) biosynthesis module, was perfectly correlated with the presence of the class D beta-lactamase gene. CMP-KDO catalyzes a key reaction in lipopolysaccharide biosynthesis (36). Further inspection of the KEGG annotations revealed that only one gene from this module was present in *C. difficile*: the arabinose-5-phosphate isomerase gene. This gene typically occurs in Gram-negative bacteria, where it plays a role in synthesis of lipopolysaccharide for the outer membrane (37), and yet a recent study identified arabinose-5-phosphate isomerase in a Gram-positive organism, *Clostridium tetani* (38). Although the function of this gene in Gram-positive bacteria is unknown, it is hypothesized to be a regulator and may modulate carbohydrate transport and metabolism (38). If so, *C. difficile* (Gram-positive) strains with arabinose-5-phosphate isomerase may have a competitive advantage because they are able to rapidly respond to the availability of the carbohydrates that are abundant in formula. It is also possible that other, potentially unknown genes are responsible for the observed effect and that these genes may not necessarily relate to metabolism of compounds in formula. Breast-fed babies have an increased abundance of *Bifidobacterium* (Text S1) (16), so the ways in which different strains of *C. difficile* interact and compete with *Bifidobacterium* may contribute to the observed trend.

### Major facilitator superfamily (MFS) pumps are associated with increased replication.

**FIG 3:**
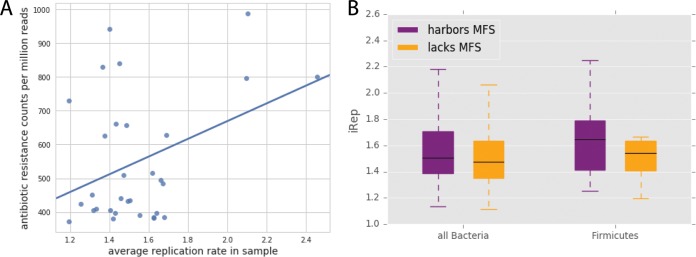
Antibiotic resistance and replication. (A) Among the samples taken within 5 days after antibiotic treatment, the antibiotic resistance potential of each sample is correlated with its mean replication index value (Pearson’s *r* = 0.39, *P* = 0.03). (B) In infants that did not receive antibiotics after the first week of life, bacteria harboring at least one major facilitator superfamily (MFS) transporter gene had significantly higher iRep values (Mann-Whitney *U* = 827,176.0, *P* = 1.55 × 10^−5^), and this pattern is apparent within the members of the *Firmicutes* phylum (Mann-Whitney *U* = 136,756.0, *P* = 0.0002).

A previous analysis revealed that antibiotic administration is associated with elevated bacterial replication index (iRep) values, which was hypothesized to be due to high resource availability after elimination of antibiotic-susceptible strains (39). Expanding upon this result, we show here that the mean iRep value for a sample in the days following antibiotic treatment is positively correlated with total resistance gene content (*P* < 0.05) (Fig. 3A). To be present in the period following antibiotic administration, all organisms must be antibiotic resistant; it is thus unclear why a larger inventory of resistance genes should lead to higher growth rates.

To characterize the effect of antibiotic resistance genes on iRep values in isolation from the confounding effects of antibiotics, we studied infants that did not receive any antibiotics after the first week of life. In these infants, organisms carrying genes for MFS transporters had significantly higher iRep values than those that do not have MFS genes (*P* < 5 × 10^−5^) (Fig. 3B). As there are known differences in median iRep values among phyla (39), the comparison was repeated within each phylum that contained members with and without MFS genes. The trend of higher iRep values for organisms with MFS was most apparent in *Firmicutes* (*P* < 5 × 10^−4^) (Fig. 3B). The genomes lacking MFS show comparatively high completeness scores (Table S3 and Text S1), suggesting that this finding is not due to missed detection of the MFS genes. Therefore, the presence of these antibiotic resistance genes appears to inherently increase replication, even when no antibiotics are being administered. This could be due to protection from antibiotics being produced at a low level by other gut organisms (40) or a result of naturally beneficial physiological functions of MFS pumps (41). We also acknowledge that this finding may simply reflect a high incidence of organisms with MFS genes present during periods of fast replication without a causal link.

### A model that predicts an organism’s response to vancomycin and cephalosporins.

We modeled the relationship between the gene content of a gut organism and its direction of change in relative abundance (increase versus decrease) after a premature infant is administered a combination of a glycopeptide antibiotic (vancomycin) and a beta-lactam antibiotic (cephalosporin [either cefotaxime or cefazolin]). Principal-component analysis (PCA) was performed on Resfams (29) and KEGG (42) annotations to generate a low-dimensional representation of each organism’s metabolic potential and resistance potential. The first five principal components (PCs) cumulatively explained 48% of the variation in the data set. Using these PCs as input, the AdaBoost-SAMME algorithm (43) was applied, with decision tree classifiers as base estimators. The model, trained on 70% of the data, performed extremely well on the validation set, with a precision value of 1.0 and a recall value of 1.0, indicating that every genome was correctly classified. Because the validation set was utilized for testing during the preliminary stages of model development, the model was also evaluated with a final test set, with which it achieved 0.9 precision and 0.7 recall (Text S1).

**FIG 4:**
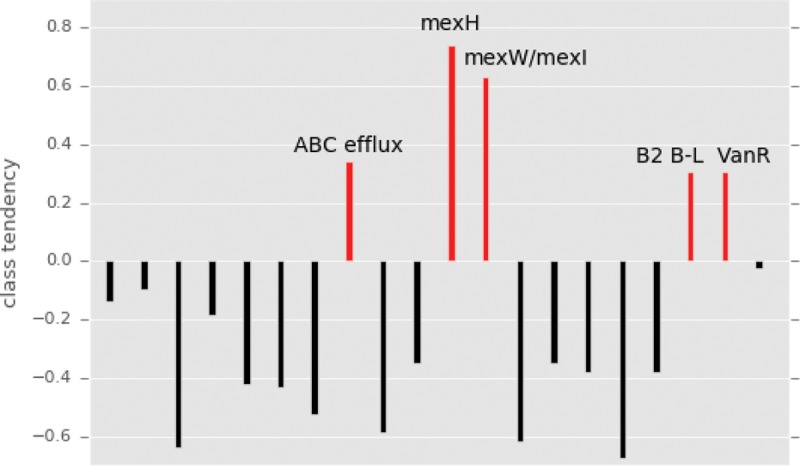
The tendency of genes to occur in the class of genomes that increased in relative abundance after antibiotics. Genes and modules strongly contributing to the principal components used in the machine learning model were identified, and class tendency was calculated using the ratio of the gene’s prevalence in the increased-abundance group to its prevalence in the decreased-abundance group. Genes associated with the increased-abundance class of genomes are colored red.

Of the features that acted as the strongest contributors to each of the PCs, five genes with a tendency to occur in microbes that increase in relative abundance after antibiotic treatment were identified (Fig. 4). One of these is the subclass B2 beta-lactamase gene, which is carried by several of the organisms that persisted after antibiotics, including *Enterococcus faecalis*, *Clostridium baratii*, and *Bradyrhizobium* sp. (Table S5 and S7 and Text S1). Subclass B2 beta-lactamase has been shown to hydrolyze carbapenems and to display much lower levels of resistance to cephalosporins (44). Considering its substrate specificity for carbapenems, this beta-lactamase may not directly contribute to an organism’s ability to persist after treatment with cephalosporins; rather, it may be linked to other, potentially unknown genes. However, the substrate specificity of an antibiotic resistance gene can depend on the organismal context of that gene (45), and a single base substitution in a beta-lactamase gene can alter substrate specificity (46), so the possibility that beta-lactamases falling into the B2 subclass may confer resistance to cephalosporins in some gut organisms should not be discounted.

Furthermore, our model shows that a gene linked to vancomycin resistance, *vanR*, is among the genes predictive of an organism’s propensity to increase in relative abundance after antibiotic treatment (Fig. 4). VanR is the transcriptional activator of an operon harboring genes involved in peptidoglycan modification (VanH, VanA, and VanX), which prevents vancomycin from binding to its target (47). This gene cluster usually resides on plasmids (48, 49). VanR, encoded by a gene that is essential for the initiation of the activity of the vancomycin resistance operon promoter (50), was chromosomally present in several genomes of organisms that increased in abundance after treatment with antibiotics, such as *Enterococcus faecalis* and *Clostridium perfringens* (Table S5 and S7 and Text S1). Because our genomic analysis precluded the assignment of genes on plasmids, VanR was the best indicator of resistance.

In addition to genes specifically encoding resistance to beta-lactams or glycopeptides, efflux pumps and transporters were also strong contributors to the PCs used as input to the model. Mex genes (of the resistance-nodulation-cell division family of drug efflux pumps) and ATP-binding cassette (ABC) transporter genes were associated with microbes that increase in relative abundance after treatment with antibiotics (Fig. 4). Multidrug efflux pumps are essential for the intrinsic drug resistance of many bacteria, and overexpression of the genes for these pumps leads to elevated resistance levels (51). *Bacteroides ovatus* and *Bacteroides helcogenes* carried multiple copies of Mex efflux pumps, while *Enterococcus faecalis* and *Clostridium baratii* harbored several ABC transporter genes (Table S5 and S7 and Text S1). Although the genomes of these organisms also encoded target-specific resistance genes such as the subclass B2 beta-lactamase, the more general pumps and transporters likely enhanced their ability to flourish after antibiotic treatment.

Previous studies have utilized data from 16S rRNA gene amplicon sequencing or read-based metagenomics of the human microbiome to predict life events and disease states of the human host using machine learning or other modeling techniques (52, 53). However, read-based metagenomics lacks resolution at the genomic level, and, due to strain-level differences in antibiotic resistance (54), taxonomy data from marker gene studies cannot be used to predict how particular organisms in a community will respond to antibiotics. Here, for the first time, we utilized the data obtained by reconstructing genomes from metagenomes to make predictions about the future states of individual gut microbes. This has tremendous potential for application in the fields of medicine and microbial ecology. For example, such a model can be used before administering drugs to a patient to verify that a particular combination of antibiotics will not lead to overgrowth of an undesirable microbe. Our report serves as a proof of concept for this application of machine learning used in conjunction with genome-resolved metagenomics to derive biological insight.

## MATERIALS AND METHODS

### Sample collection, sequencing, assembly, and gene prediction.

Fecal samples were collected from 107 infants that resided in the neonatal intensive care unit (NICU) at the Magee-Women’s Hospital in Pittsburgh, PA, during the sampling period. Briefly, DNA was extracted using a PowerSoil DNA isolation kit (Mo Bio Laboratories, Carlsbad, CA) and sequenced using an Illumina HiSeq platform. Details on sample recovery, extraction, library preparation, and sequencing have been previously reported (55–57). Using default parameters for all the programs, the reads were trimmed with Sickle (https://github.com/najoshi/sickle), cleared of human contamination following mapping to the human genome with Bowtie2 (58), and assembled with idba_ud (59). Additionally, idba_ud was used to generate coassemblies for each infant by simultaneously assembling all the samples belonging to the infant. Prodigal (60) run in the metagenomic mode was used for gene prediction.

### Genome recovery and calculation of relative abundances.

For each infant, reads from all samples from that infant were mapped to all individual assemblies from that infant as well as to the coassembly for the infant using SNAP (61). Coverage of scaffolds was calculated and used to run concoct (62) with default parameters on all individual assemblies and coassemblies. To remove redundant bins, all bins recovered from each infant were dereplicated using dRep (63) v0.4.0 with the following command: *dRep dereplicate_wf*--*S_algorithm gANI -comp 50 -con 25 -str 25 -l 50000 -pa 0.9 -nc.1*.

Using Bowtie2 (58), the reads from each sample were mapped to the set of genomes that were recovered from that particular infant. The read mapping output files were used to calculate the average coverage of each genome in each sample, and the coverage values were converted to relative abundance values by utilizing the read length, total number of reads in the sample, and genome length.

### iRep calculation.

For each sample, a set of representative genomes was first chosen from the complete collection of dereplicated genomes. First, all genomes were clustered at 98% average nucleotide identity (ANI) using dRep (63). A pangenome was then generated for each of these clusters using PanSeq (64), creating a list of fragments representing the entire sequence space of each cluster. All pangenomes of all clusters were merged, and reads from all samples were mapped to the resulting pangenome set using SNAP (61). By analyzing the coverage of all fragments in the pangenome set, the breadth of each genome in each sample was calculated (number of genome fragments >1 × coverage/total genome fragments). Genomes with less than 85% breadth were removed from analysis. For all remaining genomes, the genome from each cluster with the highest breadth was added to that sample’s representative genome list.

Next, reads from each sample were mapped to its representative genome list using bowtie2 (58) default parameters. iRep (39) was run on the resulting mapping files using default parameters and without GC correction. Only values that passed iRep’s default filtering and were <3 were considered for analysis.

### Annotation.

The amino acid sequences of genes predicted by the metaProdigal gene finding algorithm (60) were searched against Resfams (29), an antibiotic resistance gene-specific profile hidden Markov model (HMM) database, using the *hmmscan* function of HMMER v 3.1b2 (65). The --*cut_ga* option was used to set the reporting and inclusion limits as the profile-specific gathering threshold, and those limits have been manually optimized on a profile-by-profile basis to ensure Resfams prediction accuracy (29). The Resfams annotation output and the coverage of each scaffold that had a hit to a Resfams profile were used to generate sample resistance gene summaries. Each sample resistance gene summary (see Table S8 in the supplemental material), which represents the antibiotic resistance potential of a particular infant gut microbiome at a particular point in time, displays the counts per million reads (CPM) for each of the 170 antibiotic resistance gene families in the Resfams database. Additionally, genome resistance gene profiles that indicated the count of each resistance gene were developed for each genome (Table S5). Information about the database, including descriptions of the antibiotic resistance genes represented by each accession code, is available at http://www.dantaslab.org/resfams/.

10.1128/mSystems.00123-17.9 TABLE S8  Sample resistance gene summaries display the counts per million reads (CPM) for each resistance gene family in the Resfams database, for each of the 902 samples. Download TABLE S8, XLS file, 1.4 MB. Copyright © 2018 Rahman et al. 2018 Rahman et al. This content is distributed under the terms of the Creative Commons Attribution 4.0 International license.

To gather general metabolism data, all binned sequences were searched against the Kyoto Encyclopedia of Genes and Genomes (KEGG) (42) HMMs and the results were parsed for genome profiling. This resulted in a KEGG metabolism profile for each organism that displayed the fraction of each KEGG module encoded by that genome (Table S9).

10.1128/mSystems.00123-17.10 TABLE S9  The KEGG pathway module completeness of each genome. Download TABLE S9, XLS file, 1.2 MB. Copyright © 2018 Rahman et al. 2018 Rahman et al. This content is distributed under the terms of the Creative Commons Attribution 4.0 International license.

### Statistical and computational analysis.

To evaluate the effect of feeding regimen, delivery mode, gender, maternal antibiotics, and the infant’s current antibiotic therapy, three cross-sectional PERMANOVA (66) tests were performed for weeks 2, 4, and 6 using the *adonis2* function of the *vegan* package in R (67). For each infant, the first sample of each week was identified and the resistance gene summary of that sample was included in the PERMANOVA. If antibiotics were being administered on the day of sampling (which also indicates a current disease diagnosis), the infant was labeled as currently receiving antibiotics. Infants that were exclusively fed breast milk and infants that were given breast milk at any point were both classified as receiving breast milk. The Bray-Curtis dissimilarity metric was used and 9,999 permutations were performed to assess the marginal effects of the terms. The factor revealed to correspond to significant differences in antibiotic resistance gene content (*P* < 0.05) was selected for continued analysis. To identify antibiotic resistance genes associated with either formula feeding or breast milk feeding during the weeks indicated by the PERMANOVA results, the infant’s diet was used to classify sample resistance gene summaries using random forest models (68). Mann-Whitney *U* tests were performed on Resfams genes that had feature importance scores above 0.07 in the random forest model, as calculated by the Gini importance metric. Genomes containing resistance genes significantly associated with a particular feeding type, along with genomes of the same species lacking these genes, were further investigated. The ribosomal protein S3 (RPS3) genes for each genome were identified using rp16.py (https://github.com/christophertbrown/bioscripts/blob/master/bin/rp16.py). The RPS3 nucleotide sequences were aligned with MUSCLE (69) using default parameters, and a maximum-likelihood phylogenetic tree was built with RAxML (70). Pairwise Pearson correlations of Resfams data with KEGG modules within these genomes were calculated.

The Pearson correlation of the mean replication index (iRep) value for a sample and the sample’s total resistance gene content was determined for samples collected within 5 days following antibiotic treatment. The rates of replication of organisms harboring antibiotic resistance genes were compared to those of organisms lacking resistance genes of the same category. All *P* values were subjected to Bonferroni correction for multiple testing.

Infants from whom a sample was taken both before and after a post-week antibiotic treatment were identified, and the before-treatment and after-treatment samples were selected (no samples were available from the period prior to the time at which the empirical antibiotics were administered during the first week). Genomes from the selected samples were identified and labeled as either increasing or decreasing in relative abundance from the preantibiotic sample to the postantibiotic sample. Using scikit-learn (68), development of a machine learning model to predict the direction of change in relative abundance for each genome based on its Resfams and KEGG metabolism data was attempted, and yet an adequate model could not be developed, presumably due to variations in the ways in which organisms respond to different antibiotic combinations. Therefore, the data set was narrowed to include the six infants that received either cefotaxime or cefazolin (both cephalosporin antibiotics) in conjunction with vancomycin. Seventy percent of the genomes obtained from these infant samples were used for training, 15% were used as a validation set for model improvement, and 15% were held out as a final test set. Several attempts to improve model performance through algorithm choice, feature engineering, and parameter tuning were applied, and the model that exhibited the best results with regard to precision and recall was selected. This model was then used to make predictions for the final test set. Each feature constructed for the model was a principal component of the Resfams and KEGG metabolic data, and the genes/modules contributing most strongly to each of these principal components were identified. The tendency of each of the genes and modules to occur in the increased-abundance class was calculated by adding −1 to the gene’s mean value in the increased-abundance class divided by its mean value in the decreased-abundance class.

### Data availability.

The dataset used was comprised of 597 previously reported samples (55–57) and 305 new samples. These samples are available at NCBI under accession number SRP114966. The code for the analysis, along with all the data and metadata used in the analysis, is hosted at https://github.com/SumayahR/antibiotic-resistance.

## References

[1] Goossens H, Ferech M, Vander Stichele R, Elseviers M; ESAC Project Group 2005 Outpatient antibiotic use in Europe and association with resistance: a cross-national database study. Lancet 365:579–587. doi:10.1016/S0140-6736(05)17907-0. 15708101

[2] Spellberg B, Guidos R, Gilbert D, Bradley J, Boucher HW, Scheld WM, Bartlett JG, Edwards J, Infectious Diseases Society of America 2008 The epidemic of antibiotic-resistant infections: a call to action for the medical community from the Infectious Diseases Society of America. Clin Infect Dis 46:155–164. doi:10.1086/524891. 18171244

[3] Penders J, Stobberingh EE, Savelkoul PHM, Wolffs PFG 2013 The human microbiome as a reservoir of antimicrobial resistance. Front Microbiol 4:87. doi:10.3389/fmicb.2013.00087. 23616784 PMC3627978

[4] Den van den Braak N, Van Belkum A, Van Keulen M, Vliegenthart J, Verbrugh HA, Endtz HP 1998 Molecular characterization of vancomycin-resistant enterococci from hospitalized patients and poultry products in the Netherlands. J Clin Microbiol 36:1927–1932. 9650938 10.1128/jcm.36.7.1927-1932.1998 PMC104954

[5] Teuber M, Meile L, Schwarz F 1999 Acquired antibiotic resistance in lactic acid bacteria from food. Antonie van Leeuwenhoek 76:115–137. doi:10.1023/A:1002035622988. 10532375

[6] Simonsen GS, Lvseth A, Dahl KH, Kruse H 1998 Transmission of VanA-type vancomycin-resistant enterococci and vanA resistance elements between chicken and humans at avoparcin-exposed farms. Microb Drug Resist 4:313–318. doi:10.1089/mdr.1998.4.313. 9988050

[7] Langdon A, Crook N, Dantas G 2016 The effects of antibiotics on the microbiome throughout development and alternative approaches for therapeutic modulation. Genome Med 8:39. doi:10.1186/s13073-016-0294-z. 27074706 PMC4831151

[8] Clark RH, Bloom BT, Spitzer AR, Gerstmann DR 2006 Reported medication use in the neonatal intensive care unit: data from a large national data set. Pediatrics 117:1979–1987. doi:10.1542/peds.2005-1707. 16740839

[9] Faith JJ, Guruge JL, Charbonneau M, Subramanian S, Seedorf H, Goodman AL, Clemente JC, Knight R, Heath AC, Leibel RL, Rosenbaum M, Gordon JI 2013 The long-term stability of the human gut microbiota. Science 341:1237439. doi:10.1126/science.1237439. 23828941 PMC3791589

[10] Greenwood C, Morrow AL, Lagomarcino AJ, Altaye M, Taft DH, Yu Z, Newburg DS, Ward DV, Schibler KR 2014 Early empiric antibiotic use in preterm infants is associated with lower bacterial diversity and higher relative abundance of Enterobacter. J Pediatr 165:23–29. doi:10.1016/j.jpeds.2014.01.010. 24529620 PMC4074569

[11] Arboleya S, Sánchez B, Milani C, Duranti S, Solís G, Fernández N, de los Reyes-Gavilán CG, Ventura M, Margolles A, Gueimonde M 2015 Intestinal microbiota development in preterm neonates and effect of perinatal antibiotics. J Pediatr 166:538–544. doi:10.1016/j.jpeds.2014.09.041. 25444008

[12] Tanaka S, Kobayashi T, Songjinda P, Tateyama A, Tsubouchi M, Kiyohara C, Shirakawa T, Sonomoto K, Nakayama J 2009 Influence of antibiotic exposure in the early postnatal period on the development of intestinal microbiota. FEMS Immunol Med Microbiol 56:80–87. doi:10.1111/j.1574-695X.2009.00553.x. 19385995

[13] Jernberg C, Löfmark S, Edlund C, Jansson JK 2007 Long-term ecological impacts of antibiotic administration on the human intestinal microbiota. ISME J 1:56–66. doi:10.1038/ismej.2007.3. 18043614

[14] Gibson MK, Wang B, Ahmadi S, Burnham CA, Tarr PI, Warner BB, Dantas G 2016 Developmental dynamics of the preterm infant gut microbiota and antibiotic resistome. Nat Microbiol 1:16024. doi:10.1038/nmicrobiol.2016.24. 27572443 PMC5031140

[15] Penders J, Vink C, Driessen C, London N, Thijs C, Stobberingh EE 2005 Quantification of Bifidobacterium spp., Escherichia coli and Clostridium difficile in faecal samples of breast-fed and formula-fed infants by real-time PCR. FEMS Microbiol Lett 243:141–147. doi:10.1016/j.femsle.2004.11.052. 15668012

[16] Costello EK, Stagaman K, Dethlefsen L, Bohannan BJM, Relman DA 2012 The application of ecological theory toward an understanding of the human microbiome. Science 336:1255–1262. doi:10.1126/science.1224203. 22674335 PMC4208626

[17] Yassour M, Vatanen T, Siljander H, Hämäläinen AM, Härkönen T, Ryhänen SJ, Franzosa EA, Vlamakis H, Huttenhower C, Gevers D, Lander ES, Knip M; DIABIMMUNE Study Group, Xavier RJ 2016 Natural history of the infant gut microbiome and impact of antibiotic treatment on bacterial strain diversity and stability. Sci Transl Med 8:343ra81. doi:10.1126/scitranslmed.aad0917. PMC5032909 27306663

[18] Wampach L, Heintz-Buschart A, Hogan A, Muller EEL, Narayanasamy S, Laczny CC, Hugerth LW, Bindl L, Bottu J, Andersson AF, de Beaufort C, Wilmes P 2017 Colonization and succession within the human gut microbiome by archaea, bacteria and microeukaryotes during the first year of life. Front Microbiol 8:738. doi:10.3389/fmicb.2017.00738. 28512451 PMC5411419

[19] Penders J, Thijs C, Vink C, Stelma FF, Snijders B, Kummeling I, van den Brandt PA, Stobberingh EE 2006 Factors influencing the composition of the intestinal microbiota in early infancy. Pediatrics 118:511–521. doi:10.1542/peds.2005-2824. 16882802

[20] Stewart CJ, Embleton ND, Clements E, Luna PN, Smith DP, Fofanova TY, Nelson A, Taylor G, Orr CH, Petrosino JF, Berrington JE, Cummings SP 2017 Cesarean or vaginal birth does not impact the longitudinal development of the gut microbiome in a cohort of exclusively preterm infants. Front Microbiol 8:1–9. doi:10.3389/fmicb.2017.01008. 28634475 PMC5459931

[21] Chu DM, Ma J, Prince AL, Antony KM, Seferovic MD, Aagaard KM 2017 Maturation of the infant microbiome community structure and function across multiple body sites and in relation to mode of delivery. Nat Med 23:314–326. doi:10.1038/nm.4272. 28112736 PMC5345907

[22] Cong X, Xu W, Janton S, Henderson WA, Matson A, McGrath JM, Maas K, Graf J 2016 Gut microbiome developmental patterns in early life of preterm infants: impacts of feeding and gender. PLoS One 11:e0152751. doi:10.1371/journal.pone.0152751. 27111847 PMC4844123

[23] Mshvildadze M, Neu J, Shuster J, Theriaque D, Li N, Mai V 2010 Intestinal microbial ecology in premature infants assessed with non-culture-based techniques. J Pediatr 156:20–25. doi:10.1016/j.jpeds.2009.06.063. 19783002 PMC3628625

[24] Keski-nisula L, Kyynäräinen HR, Kärkkäinen U, Karhukorpi J, Heinonen S, Pekkanen J 2013 Maternal intrapartum antibiotics and decreased vertical transmission of Lactobacillus to neonates during birth. Acta Paediatr 102:480–485. doi:10.1111/apa.12186. 23398392

[25] Fouhy F, Guinane CM, Hussey S, Wall R, Ryan CA, Dempsey EM, Murphy B, Ross RP, Fitzgerald GF, Stanton C, Cotter PD 2012 High-throughput sequencing reveals the incomplete, short-term recovery of infant gut microbiota following parenteral antibiotic treatment with ampicillin and gentamicin. Antimicrob Agents Chemother 56:5811–5820. doi:10.1128/AAC.00789-12. 22948872 PMC3486619

[26] Glass HC, Andrew CT, Stayer SA, Brett C, Cladis F, Davis PJ 2015 Outcomes for extremely premature infants. Anesth Analg 120:1337–1351. doi:10.1213/ANE.0000000000000705. 25988638 PMC4438860

[27] Underwood MA, Sohn K 2017 The microbiota of the extremely preterm infant. Clin Perinatol 44:407–427. doi:10.1016/j.clp.2017.01.005. 28477669 PMC6361543

[28] Schwiertz A, Gruhl B, Löbnitz M, Michel P, Radke M, Blaut M 2003 Development of the intestinal bacterial composition in hospitalized preterm infants in comparison with breast-fed, full-term infants. Pediatr Res 54:393–399. doi:10.1203/01.PDR.0000078274.74607.7A. 12788986

[29] Gibson MK, Forsberg KJ, Dantas G 2015 Improved annotation of antibiotic resistance determinants reveals microbial resistomes cluster by ecology. ISME J 9:207–216. doi:10.1038/ismej.2014.106. 25003965 PMC4274418

[30] Poole K 2014 Stress responses as determinants of antimicrobial resistance in Pseudomonas aeruginosa: multidrug efflux and more. Can J Microbiol 60:783–791. doi:10.1139/cjm-2014-0666. 25388098

[31] Poole K 2008 Bacterial multidrug efflux pumps serve other functions. Microbe 3:179–185. doi:10.1128/microbe.3.179.1.

[32] Nagayama K, Fujita K, Takashima Y, Ardin AC, Ooshima T, Matsumoto-Nakano M 2014 Role of ABC transporter proteins in stress responses of Streptococcus mutans. Oral Heal Dent Manag 13:359–365. 24984648

[33] Anderson MJ 2001 A new method for non-parametric multivariate analysis of variance. Austral Ecol 26:32–46. doi:10.1111/j.1442-9993.2001.01070.pp.x.

[34] Anderson MJ, Walsh DCI 2013 PERMANOVA, ANOSIM, and the Mantel test in the face of heterogeneous dispersions: what null hypothesis are you testing? Ecol Monogr 83:557–574. doi:10.1890/12-2010.1.

[35] Szarecka A, Lesnock KR, Ramirez-Mondragon CA, Nicholas HB, Wymore T 2011 The Class D beta-lactamase family: residues governing the maintenance and diversity of function. Protein Eng Des Sel 24:801–809. doi:10.1093/protein/gzr041. 21859796 PMC3170078

[36] Wang X, Quinn PJ 2010 Lipopolysaccharide: biosynthetic pathway and structure modification. Prog Lipid Res 49:97–107. doi:10.1016/j.plipres.2009.06.002. 19815028

[37] Meredith TC, Aggarwal P, Mamat U, Lindner B, Woodard RW 2006 Redefining the requisite lipopolysaccharide structure in Escherichia coli. ACS Chem Biol 1:33–42. doi:10.1021/cb0500015. 17163638

[38] Cech DL, Markin K, Woodard RW 2017 Identification of a d-arabinose-5-phosphate isomerase in the Gram-positive Clostridium tetani. J Bacteriol 199. doi:10.1128/JB.00246-17. PMC5553037 28630128

[39] Brown CT, Olm MR, Thomas BC, Banfield JF 2016 Measurement of bacterial replication rates in microbial communities. Nat Biotechnol 34:1256–1263. doi:10.1038/nbt.3704. 27819664 PMC5538567

[40] Modi SR, Collins JJ, Relman DA 2014 Antibiotics and the gut microbiota. J Clin Invest 124:4212–4218. doi:10.1172/JCI72333. 25271726 PMC4191029

[41] Piddock LJV 2006 Multidrug-resistance efflux pumps — not just for resistance. Nat Rev Microbiol 4:629–636. doi:10.1038/nrmicro1464. 16845433

[42] Kanehisa M, Goto S 2000 KEGG: Kyoto encyclopedia of genes and genomes. Nucleic Acids Res 28:27–30. doi:10.1093/nar/28.1.27. 10592173 PMC102409

[43] Hastie T, Rosset S, Zhu J, Zou H 2009 Multi-class AdaBoost. Stat Interface 2:349–360. doi:10.4310/SII.2009.v2.n3.a8.

[44] Hernandez Valladares MH, Felici A, Weber G, Adolph HW, Zeppezauer M, Rossolini GM, Amicosante G, Frère JM, Galleni M 1997 Zn(II) dependence of the Aeromonas hydrophila AE036 metallo-beta-lactamase activity and stability. Biochemistry 36:11534–11541. doi:10.1021/bi971056h. 9298974

[45] Hansen LH, Jensen LB, Sørensen HI, Sørensen SJ 2007 Substrate specificity of the OqxAB multidrug resistance pump in Escherichia coli and selected enteric bacteria. J Antimicrob Chemother 60:145–147. doi:10.1093/jac/dkm167. 17526501

[46] Jacoby GA, Medeiros AA 1991 More extended-spectrum beta-lactamases. Antimicrob Agents Chemother 35:1697–1704. doi:10.1128/AAC.35.9.1697. 1952834 PMC245253

[47] Hughes D 2003 Exploiting genomics, genetics and chemistry to combat antibiotic resistance. Nat Rev Genet 4:432–441. doi:10.1038/nrg1084. 12776213

[48] Boyce JM 1997 Vancomycin-resistant enterococcus. Detection, epidemiology and control measures. Infect Dis Clin North Am 11:367–384. doi:10.1016/S0891-5520(05)70361-5. 9187952

[49] Périchon B, Courvalin P 2009 VanA-type vancomycin-resistant Staphylococcus aureus. Antimicrob Agents Chemother 53:4580–4587. doi:10.1128/AAC.00346-09. 19506057 PMC2772335

[50] Arthur M, Quintiliani R 2001 Regulation of VanA- and VanB-type glycopeptide resistance in enterococci. Antimicrob Agents Chemother 45:375–381. doi:10.1128/AAC.45.2.375-381.2001. 11158729 PMC90301

[51] Li XZ, Nikaido H 2009 Efflux-mediated drug resistance in bacteria: an update. Drugs 69:1555–1623. doi:10.2165/11317030-000000000-00000. 19678712 PMC2847397

[52] DiGiulio DB, Callahan BJ, McMurdie PJ, Costello EK, Lyell DJ, Robaczewska A, Sun CL, Goltsman DSA, Wong RJ, Shaw G, Stevenson DK, Holmes SP, Relman DA 2015 Temporal and spatial variation of the human microbiota during pregnancy. Proc Natl Acad Sci U S A 112:11060–11065. doi:10.1073/pnas.1502875112. 26283357 PMC4568272

[53] Yazdani M, Taylor BC, Debelius JW, Li W, Knight R, Smarr L 2016 Using machine learning to identify major shifts in human gut microbiome protein family abundance in disease, p 1273–1280. *In* IEEE International Conference on Big Data, Washington, DC.

[54] Kumar V, Sun P, Vamathevan J, Li Y, Ingraham K, Palmer L, Huang J, Brown JR 2011 Comparative genomics of Klebsiella pneumoniae strains with different antibiotic resistance profiles. Antimicrob Agents Chemother 55:4267–4276. doi:10.1128/AAC.00052-11. 21746949 PMC3165360

[55] Raveh-Sadka T, Thomas BC, Singh A, Firek B, Brooks B, Castelle CJ, Sharon I, Baker R, Good M, Morowitz MJ, Banfield JF 2015 Gut bacteria are rarely shared by co-hospitalized premature infants, regardless of necrotizing enterocolitis development. Elife 4:e05477. doi:10.7554/eLife.05477. PMC4384745 25735037

[56] Raveh-Sadka T, Firek B, Sharon I, Baker R, Brown CT, Thomas BC, Morowitz MJ, Banfield JF 2016 Evidence for persistent and shared bacterial strains against a background of largely unique gut colonization in hospitalized premature infants. ISME J 10:2817–2830. doi:10.1038/ismej.2016.83. 27258951 PMC5148203

[57] Brooks B, Olm MR, Firek BA, Baker R, Thomas BC, Morowitz MJ, Banfield JF 27 11 2017 Strain-resolved analysis of the hospital room and hospitalized infants reveals overlap between the human and room microbiome. Nat Commun doi:10.1038/s41467-017-02018-w. PMC5703836 29180750

[58] Langmead B, Salzberg SL 2012 Fast gapped-read alignment with Bowtie 2. Nat Methods 9:357–359. doi:10.1038/nmeth.1923. 22388286 PMC3322381

[59] Peng Y, Leung HCM, Yiu SM, Chin FYL 2012 IDBA-UD: a de novo assembler for single-cell and metagenomic sequencing data with highly uneven depth. Bioinformatics 28:1420–1428. doi:10.1093/bioinformatics/bts174. 22495754

[60] Hyatt D, Chen GL, Locascio PF, Land ML, Larimer FW, Hauser LJ 2010 Prodigal: prokaryotic gene recognition and translation initiation site identification. BMC Bioinformatics 11:119. doi:10.1186/1471-2105-11-119. 20211023 PMC2848648

[61] Zaharia M, Bolosky WJ, Curtis K, Fox A, Patterson D, Shenker S, Stoica I, Karp RM, Sittler T 2011 Faster and more accurate sequence alignment with SNAP. arXiv https://arxiv.org/abs/1111.5572.

[62] Alneberg J, Bjarnason BS, De Bruijn I, Schirmer M, Quick J, Ijaz UZ, Lahti L, Loman NJ, Andersson AF, Quince C 2014 Binning metagenomic contigs by coverage and composition. Nat Methods 11:1144–1146. doi:10.1038/nmeth.3103. 25218180

[63] Olm MR, Brown CT, Brooks B, Banfield JF 2017 dRep: a tool for fast and accurate genome de-replication that enables tracking of microbial genotypes and improved genome recovery from metagenomes. ISME J 11:2864–2868. doi:10.1038/ismej.2017.126. 28742071 PMC5702732

[64] Laing C, Buchanan C, Taboada EN, Zhang Y, Kropinski A, Villegas A, Thomas JE, Gannon VPJ 2010 Pan-genome sequence analysis using Panseq: an online tool for the rapid analysis of core and accessory genomic regions. BMC Bioinformatics 11:461. doi:10.1186/1471-2105-11-461. 20843356 PMC2949892

[65] Finn RD, Clements J, Eddy SR 2011 HMMER web server: interactive sequence similarity searching. Nucleic Acids Res 39:W29–W37. doi:10.1093/nar/gkr367. 21593126 PMC3125773

[66] McArdle BH, Anderson MJ 2001 Fitting multivariate models to community data: a comment on distance-based redundancy analysis. Ecology 82:290–297. doi:10.1890/0012-9658(2001)082[0290:FMMTCD]2.0.CO;2.

[67] Dixon P 2003 VEGAN, a package of R functions for community ecology. J Veg Sci 14:927–930. doi:10.1111/j.1654-1103.2003.tb02228.x.

[68] Pedregosa F, Varoquaux G, Gramfort A, Michel V, Thirion B, Grisel O, Blondel M, Prettenhofer P, Weiss R, Dubourg V, Vanderplas J, Passos A, Cournapeau D, Brucher M, Perrot M, Duchesnay É 2012 Scikit-learn: machine learning in Python. J Mach Learn Res 12:2825–2830. http://www.jmlr.org/papers/v12/pedregosa11a.html.

[69] Edgar RC 2004 MUSCLE: multiple sequence alignment with high accuracy and high throughput. Nucleic Acids Res 32:1792–1797. doi:10.1093/nar/gkh340. 15034147 PMC390337

[70] Stamatakis A 2014 RAxML version 8: a tool for phylogenetic analysis and post-analysis of large phylogenies. Bioinformatics 30:1312–1313. doi:10.1093/bioinformatics/btu033. 24451623 PMC3998144

