# Supplementary material for: Machine Learning Leveraging Genomes from Metagenomes Identifies Influential Antibiotic Resistance Genes in the Infant Gut Microbiome
Source: mSystems. 2018 Jan 9;3(1):e00123-17. doi: 10.1128/mSystems.00123-17 (PMC5758725; doi:10.1128/mSystems.00123-17)
Supplement: TEXT S1 [file sys001182161s1.pdf]

antibiotic_resistance_analysis

November 6, 2017

0.1 Analysis of antibiotic resistance in the premature infant gut microbiome

0.1.1 Sumayah F. Rahman 2017

0.1.2 Load libraries

```
In [211]: import pandas as pd
          import numpy as np
          import scipy
          from scipy.stats import chi2_contingency
          import matplotlib.pyplot as plt
          import matplotlib.patches as mpatches
          import seaborn as sns
          import statsmodels
          from statsmodels import sandbox
          from statsmodels.sandbox import stats
          from statsmodels.sandbox.stats import multicomp
          import sklearn
          from sklearn import preprocessing
          from sklearn.decomposition import PCA
          from sklearn.tree import DecisionTreeClassifier
          from sklearn.ensemble import RandomForestClassifier, AdaBoostClassifier
          from sklearn.metrics import precision_recall_fscore_support as score
          import collections
          from pylab import plot, show, savefig, xlim, figure, hold, ylim, legend, boxplot, setp,
          axes
          import warnings; warnings.simplefilter('ignore')
          %load_ext rpy2.ipython
          %load_ext iversions
```

```
In [163]: %iversions
```

```
numpy       1.13.3
sklearn     0.18.1
pandas      0.21.0
statsmodels  0.6.1
scipy       1.0.0
seaborn     0.7.1

Python 3.5.2 [Mon Nov 06, 2017 09:23:53]
```

0.1.3 Load annotation data, iRep data, and metadata

We processed over 4 terrabases (Tb) of sequence data in a metagenomics pipeline to generate the data for the below analysis. The scaffold coverage file was produced by read mapping metagenome assemblies (assembled with idba_ud) to their respective short reads sequence fastq files, using bowtie2. Scaffold-to-bin file is based on Concoct genome bins. Antibiotic resistance

(Resfams) and general metabolism (KEGG) genes were annotated using hidden Markov models. Further details on how these files were produced can be found in the 'Methods' section of the associated publication. The infant metadata and sample metadata files were created by re-formatting medical data from the Magee-Women's hospital of Pittsburgh, PA.

In [212]:
```
%%bash

#Remove excess lines from Resfams HMMER output file so that it is parsable
sed -i -e 1,3d good-data-files/resfams_HMM_output.txt
head -n 104384 good-data-files/resfams_HMM_output.txt > good-data-
files/resfams_HMM_output_clean.txt
```

In [213]:
```
infant_metadata = pd.read_csv("infantMetadata_3April17.csv")
sample_metadata = pd.read_csv("sampleMetadata_3April17.csv")
project_info = pd.read_csv("project_info.csv")
scaffold_coverage = pd.read_csv("scaffold_coverage.csv")
resfams_metadata = pd.read_csv("resfams_metadata.csv")
resfams_output = pd.read_table("resfams_HMM_output_clean.txt", delimiter=r"\s+",
header=None)
KEGG_gene_annotations = pd.read_csv("genes_in_concot_bins.keggHMM.report.tab", sep="\t")
KEGG_genome_profiles =
pd.read_csv("genes_in_concot_bins.module.completeness.reliable_plus_eval_1e-10.tab", sep
="\t")
scaf2bin = pd.read_table("all.stb", header=None)
genome_taxonomy = pd.read_csv("concoct_genome_taxonomy.csv")
iRep_info = pd.read_csv("iRep_w_metadata.csv")
scaf2bin_nonderep = pd.read_table("filteredNondRep.stb", header=None)
genome_metrics = pd.read_csv("Chdb.csv")
```

0.1.4 Infant Characteristics

In [166]:
```
def label_breastmilk(row):
    """Return 'True' if the infant received breastmilk at some point during the time
period samples
    were taken, and return 'False' if the infant received formula only. """
    if row['feeding'] == 'Breast' or row['feeding'] == 'Combination':
        return(True)
    if row['feeding'] == 'Forumla':
        return(False)

def calculate_characteristics(df, category):
    """Calculate the number of infants with particular characteristics (feeding, gender,
    maternal antibiotics, birth mode.)"""
    # Using the metadata dataframe, determine the number of infants with each
characteristic
    received_breastmilk =
len(df.loc[df['received_breastmilk']==True]['infant'].unique())
    male = len(df.loc[df['gender']=='M']['infant'].unique())
    received_maternal_ab = len(df.loc[df['maternal_ab']==True]['infant'].unique())
    c_section = len(df.loc[df['birth_mode']=='C-section']['infant'].unique())
    vaginal_birth = len(df.loc[df['birth_mode']=='Vaginal']['infant'].unique())
    number_samples = len(df)
    number_infants = len(df['infant'].unique())
    # Create an ordered dictionaty that represents the categories and the values for
each category
    odict = collections.OrderedDict([('Samples', number_samples),
                                     ('Infants', number_infants),
                                     ('Male sex', male),
                                    ('Received breastmilk', received_breastmilk),
                                     ('Delivered by C-section', c_section),
                                     ('Maternal antibiotics', received_maternal_ab)])
    characteristics_df = pd.DataFrame(data=odict, index=category) # Convert dict to
dataframe
    return(characteristics_df)
```

```
def generate_characteristic_table(sample_metadata, infant_metadata):
    """Produce table that displays the infant metadata in regard to the infants that
only received
    antibiotics in the first week of life (left) and the infants that received
additional antibiotics (right)."""
    all_metadata = sample_metadata.merge(infant_metadata, on='infant') # Combine sample
and infant metadata
    all_metadata['received_breastmilk'] = all_metadata.apply(lambda row:
label_breastmilk(row),axis=1)
    extra_abx_babies = all_metadata.loc[all_metadata['postweek_ab'] == True] # babies
that received additional abx
    healthy_babies = all_metadata.loc[all_metadata['postweek_ab'] == False] # babies
that only received abx in 1st week
    df = calculate_characteristics(extra_abx_babies,['Received postweek antibiotics due
to disease'])
    df2 = df.transpose()
    df2['Category'] = df2.index
    df3 = calculate_characteristics(healthy_babies, ['No antibiotics after the first
week of life'])
    df4 = df3.transpose()
    df4['Category'] = df4.index
    df5 = df2.merge(df4, on='Category') # merge healthy-infant-df with extra-
antibiotics-infant df
    df5 = df5[['Category', 'No antibiotics after the first week of life',
               'Received postweek antibiotics due to disease']] # column order
    return(df5)

table = generate_characteristic_table(sample_metadata, infant_metadata)
table
```

Out[166]:

```
                  Category  No antibiotics after the first week of life  \
0                  Samples                                          604
1                  Infants                                           71
2                 Male sex                                           34
3      Received breastmilk                                           52
4  Delivered by C-section                                            54
5     Maternal antibiotics                                           24

   Received postweek antibiotics due to disease
0                                           298
1                                            36
2                                            17
3                                            32
4                                            22
5                                            20
```

In [167]:

```
# In addition to the binary characteristics included in the table above, let's look at
gestational age and birthweight

def label_preterm(row):
    if row['gestational_age'] >= 34 and row['gestational_age'] < 37:
        return('late preterm')
    elif row['gestational_age'] >= 32 and row['gestational_age'] < 34:
        return('moderate preterm')
    elif row['gestational_age'] >= 28 and row['gestational_age'] < 32:
        return('very preterm')
    elif row['gestational_age'] < 28:
        return('extremely preterm')

infant_metadata['prematurity'] = infant_metadata.apply(lambda row:
label_preterm(row),axis=1)
```

```
preterm_counts = infant_metadata['prematurity'].value_counts()
preterm_counts.sort_index().plot(kind='bar', width=0.8,figsize=(3,2))
plt.ylabel("# infants")
plt.xticks(rotation=20, ha='right')
plt.show()

infant_metadata['Weight (g)'].plot(kind='hist', figsize=(3,2))
plt.xlabel("birthweight (g)")
plt.show()
```

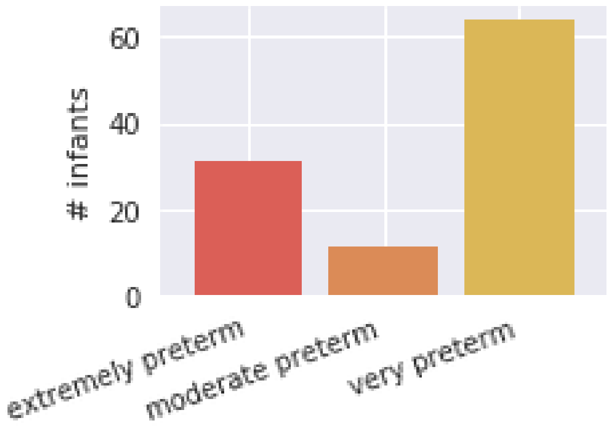

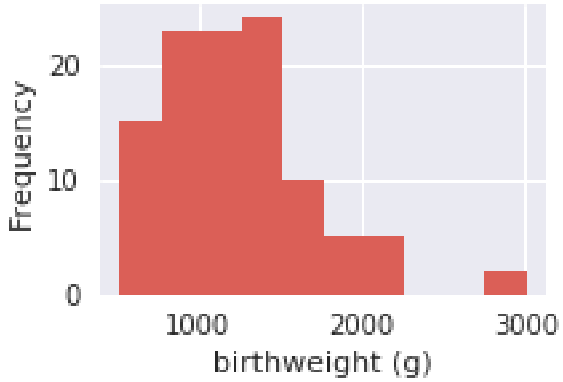

0.1.5 Sample and genome resistance gene summaries

Utilize data from Resfams HMM annotations to create sample resistance gene summaries, which represent the antibiotic resistance potential of a particular infant microbiome at a certain point in time, and genome resistance gene profiles, which represent the resistance potential of a particular organism.

```
In [168]: def filter_tblout(output_df):
              """Extract the important output
              from the HMMER -tblout output format run on Resfams.hmm"""
```

```
    output_filtered1 = output_df.iloc[:,[0,2,3]] # pull out the columns containing the
scaffold designation
                                                 # the Resfams accession number, and the
annotation description
    output_filtered2 = output_filtered1.rename(columns={0: 'scaffold', 2: 'annotation',
3: 'RF'})
    output_filtered2['scaffold'] = output_filtered2['scaffold'].apply(lambda x:
"_".join(x.split("_")[:-1]))
    return(output_filtered2)

def coverage_in_samples(filtered_df):
    """Utilize scaffold coverage data
    to generate a list of dataframes that describe the
    Resfams coverage in each sample"""
    # merge the scaffold_coverage with the resfams output
    resfams_with_coverage = filtered_df.merge(scaffold_coverage, how="inner",
on='scaffold')
    # add column to designate sample
    resfams_with_coverage['sample'] = resfams_with_coverage['scaffold'].apply(lambda x:
"_".join(x.split("_")[:-2]))
    # group annotations by sample
    l_grouped = list(resfams_with_coverage.groupby(['sample']))
    return(l_grouped)

def create_sample_res_gene_summary(list_of_coverage_dfs):
    """Generate sample resistance gene summaries using
    Resfams annotations combined with normalized coverage data"""
    # gather all possible RF accesions
    RF_accessions = resfams_metadata.iloc[:,:1]
    series_RF_accessions = RF_accessions[RF_accessions.columns[0]]
    # Calculate the normalized resistance gene presence of each sample
    sample_to_summary = {}
    for i in range(0,len(list_of_coverage_dfs)): # for each infant sample
        sample_df = list_of_coverage_dfs[i][1]
        # count the number of times each Resfam occurs
        sample_df_counted = sample_df.groupby(['RF'])['coverage'].sum().reset_index()
        for RF in series_RF_accessions:
            # add Resfam to dataframe if it is not there
            if RF not in list(sample_df_counted['RF']):
                 # make new record into dataframe
                new_RF = pd.DataFrame([[RF, 0]],columns=['RF','coverage'])
                sample_df_counted = pd.concat([sample_df_counted, new_RF])
        sample_df_sorted = sample_df_counted.sort_values(by='RF')
        # obtain raw reads for each sample
        scaffold_code = list_of_coverage_dfs[i][0]
        num_reads =
int(project_info[project_info['scaffold_code']==scaffold_code]['reads'])
        #calculate counts per million
        sample_df_sorted['normalized_coverage'] =
sample_df_sorted['coverage'].apply(lambda x: (x/num_reads)*1000000)
        sample_summary = sample_df_sorted.drop('coverage',axis=1)
        sample_to_summary[scaffold_code] = sample_summary
    # Generate sample resistance gene summaries for each sample
    list_of_individual_dfs = []
    all_scaffold_codes = list(project_info['scaffold_code'])
    all_sample_names = list(project_info['public_code'])
    for i in range(len(all_scaffold_codes)):
        # skip co-assemblies
        if all_sample_names[i].split("_")[-1] != '000G1':
            if "_".join(all_sample_names[i].split("_")[0:2]) != 'S2_CON' \
            and "_".join(all_sample_names[i].split("_")[0:2]) != 'SP_CRL':
                try:
                    transposed_summary =
sample_to_summary[all_scaffold_codes[i]].transpose()
                    summary1 = transposed_summary.reset_index()
                    summary1 = summary1.replace('normalized_coverage',
all_sample_names[i])
                    summary1.columns = summary1.iloc[0]
```

```
                    summary1 = summary1.drop(0,axis=0)
                    summary1 = summary1.rename(columns={'RF':'sample'})
                    list_of_individual_dfs.append(summary1)
                except KeyError:
                    # some samples have no annotated resistance genes
                    continue
        # combine the sample resistance gene summaries into one dataframe
        all_sample_summaries = pd.concat(list_of_individual_dfs)
        return(all_sample_summaries)

    def create_genome_res_summary(filtered_df, scaf2bin):
        scaf2bin = scaf2bin.rename(columns={0: 'scaffold', 1: 'bin'})
        # generate a dataframe that states which Resfams are annotated on each scaffold
        annot2bin = filtered_df.merge(scaf2bin, on='scaffold')
        # count resfams in each bin and create genome summaries
        RFs_in_bin = annot2bin.groupby(by=['bin','RF']).count()
        genome_resistance_summaries = RFs_in_bin.reset_index().pivot(index='bin',
    columns='RF')
        genome_resistance_summaries.columns =
    genome_resistance_summaries.columns.droplevel()
        genome_resistance_summaries = genome_resistance_summaries.fillna(value=0)
        genome_resistance_summaries =
    genome_resistance_summaries.loc[:,~genome_resistance_summaries.columns.duplicated()]
        return(genome_resistance_summaries)

    # filter Resfams annotations
    resfams_output_filtered = filter_tblout(resfams_output)

    # generate resfams coverage dataframe for each sample
    list_of_sample_dfs = coverage_in_samples(resfams_output_filtered)

    # build a dataframe of sample resistance gene summaries
    sample_resistance_gene_summaries =
    create_sample_res_gene_summary(list_of_sample_dfs).reset_index()
    # build a dataframe of genome resistance gene profiles using Resfams counts
    genome_resistance_gene_summaries = create_genome_res_summary(resfams_output_filtered,
    scaf2bin)
```

In [169]:
```
# scaf2bin_nonderep is a scaffolds to bin file for all genomes binned, not just de-
replicated set
scaffolds_in_resistome = resfams_output[0]
scaffolds_in_bins = set(scaf2bin_nonderep[0])
scafs_in_resistome = []
for scaffold in scaffolds_in_resistome:
    new_scaf = "_".join(scaffold.split("_")[:-1])
    scafs_in_resistome.append(new_scaf)
unassignable_scaffolds = set(scafs_in_resistome) - scaffolds_in_bins
fraction_unassignable = len(unassignable_scaffolds)/len(scafs_in_resistome)
print("{0:.1f} of the resistome was not assigned to any
genome.".format(fraction_unassignable))

# Calculate minimum presence in co-assembly required for a genome to be included in
analysis
co_assemblies = project_info.loc[project_info['DOL'] == 0]
mean_depth = co_assemblies['read_bases'].mean()
presence_requirement = (8*3300000 / mean_depth)*100 # 8x coverage required for assembly,
3.3 Mbp average genome size
print("We were able to recover genomes that are at least {0:.2f} of the community over
first month of life.".format \
      (presence_requirement))
```

```
0.2 of the resistome was not assigned to any genome.
We were able to recover genomes that are at least 0.07 of the community over first
month of life.
```

0.1.6 Breakdown of antibiotic resistance by mechanism

Antibiotic resistance genes have different mechanisms by which they reduce or eliminate a bacterium's susceptibility to antibiotics. Additionally, the composition of the gut microbiome is known to change during the first few months of life, so it is likely that there is variation of the resistome (resistance genes) during this time frame. This visualization is a broad overview of the infant gut resistome content over time.

```
In [170]: def correspond_metadata_and_data(resistance_df):
              """Reorders the sample resistance gene summary dataframe and the metadata dataframe
              so that they properly correspond, return both ordered dataframes"""
              # connect the samples to the infant using the sample_metadata df
              all_res_gene_summaries2 = sample_resistance_gene_summaries.merge(sample_metadata,
          on='sample')
              # connect all data using infant sheet
              all_res_gene_summaries3 = all_res_gene_summaries2.merge(infant_metadata,
          on='infant')
              # separate the data (resistance gene summaries) and the metadata
              samples = all_res_gene_summaries3['sample']
              res_gene_summaries_only = all_res_gene_summaries3.iloc[:, 2:172] # select resfams
          columns
              res_gene_summaries_only = res_gene_summaries_only.set_index(samples)
              metadata_only = all_res_gene_summaries3.iloc[:, 172:] # select metadata columns
              metadata_only['sample'] = samples
              return(res_gene_summaries_only, metadata_only)

          def convert_res_to_category(ordered_resistance_df):
              """Convert each sample from individual res genes to category,
              return dataframe of sample resistance category summaries"""
              # generate a list of mechanisms to classify each Resfam as the appropriate mechanism
          category
              list_of_RFs = ordered_resistance_df.columns[1:]
              list_of_mechanisms = []
              for RF in list_of_RFs:
                  list_of_mechanisms.append(resfams_metadata.loc[resfams_metadata['Resfam ID'] ==
          RF] \
                                                    ['Mechanism Classification'])
              new_list_of_mechanisms = []
              for mech in list_of_mechanisms:
                  mech = mech.values[0]
                  new_list_of_mechanisms.append(mech)
              ordered_resistance_df = ordered_resistance_df.drop(ordered_resistance_df.columns[0],
          axis=1)
              # rename the columns with the mechanism rather than Resfams accession
              ordered_resistance_df.columns = new_list_of_mechanisms
              # sum the Resfams belonging to the same mechanism category
              sample_res_category_summaries =
          ordered_resistance_df.groupby(ordered_resistance_df.columns, axis=1).sum()
              return(sample_res_category_summaries)

          def label_week(row):
              """Convert day to week."""
              if row['DOL'] >= 8 and row['DOL'] < 15:
                  return(2)
              elif row['DOL'] >= 15 and row['DOL'] < 22:
                  return(3)
              elif row['DOL'] >= 22 and row['DOL'] < 29:
                  return(4)
              elif row['DOL'] >= 29 and row['DOL'] < 36:
                  return(5)
              elif row['DOL'] >= 36 and row['DOL'] < 43:
                  return(6)
              elif row['DOL'] >= 43 and row['DOL'] < 50:
                  return(7)
              elif row['DOL'] >= 50 and row['DOL'] < 57:
                  return(8)
```

```
    elif row['DOL'] > 57:
        return('9+')

def make_cat_res_sum_by_week_df(sample_res_category_sum_df, boolean):
    """Group the infant samples in the sample resistance
    gene category summaries by week. Return dataframe grouped by week
    and original dataframe with DOL added"""
    metadata_ordered.index = sample_res_category_sum.index
    # narrow to babies in the correct antibiotic category (did vs did not receive
postweek)
    sample_res_category_sum_df['postweek_ab'] = metadata_ordered['postweek_ab']
    sample_res_category_sum_df =
sample_res_category_sum_df.loc[sample_res_category_sum_df['postweek_ab'] == boolean]
    sample_res_category_sum_df['DOL'] = metadata_ordered['DOL']
    # withdraw datapoints influenced by over-representation of resistant bugs
    sample_res_category_sum_df =
sample_res_category_sum_df.loc[sample_res_category_sum_df['ABC Transporter']<500]
    # convert days into weeks and group samples by infant week of life
    sample_res_category_sum_df['infant age in weeks'] =
sample_res_category_sum_df.apply(lambda row: \
 label_week(row),axis=1)
    # calculate the mean resistance mechanism cpm for each week category
    sample_res_category_sum_df =
sample_res_category_sum_df.loc[sample_res_category_sum_df['infant age in weeks'] \
                                                               != '9+']
    sample_res_cat_by_week0 = sample_res_category_sum_df.groupby('infant age in
weeks').mean()
    sample_res_cat_by_week1 = sample_res_cat_by_week0.ix[:,:-1]
    # remove any categories that are entirely zero
    sample_res_cat_by_week = sample_res_cat_by_week1.loc[:, (sample_res_cat_by_week1 !=
0).any(axis=0)]
    return(sample_res_cat_by_week, sample_res_category_sum_df)


sample_res_gene_ordered, metadata_ordered =
correspond_metadata_and_data(sample_resistance_gene_summaries)

sample_res_category_sum = convert_res_to_category(sample_res_gene_ordered)

noabx_sample_res_cat_by_week, noabx_sample_res_category_sum_df =
make_cat_res_sum_by_week_df(sample_res_category_sum, False)
# create bar graph displaying infant week and mean resistance content by category
sns.set_palette(sns.color_palette("husl", 15))
fig =  noabx_sample_res_cat_by_week.plot(kind='bar', stacked=True, width=0.75,
figsize=(8,7))
plt.ylabel('counts per million reads')
plt.title('Babies that did not receive postweek antibiotics')
handles, labels = fig.get_legend_handles_labels()
fig.legend(handles[::-1], labels[::-1], title='Resistance Mechanisms', loc='center
left', bbox_to_anchor=(1, 0.5))
plt.show()
# calculate correlation
total_counts = noabx_sample_res_category_sum_df.sum(axis=1)
pearson_r, p_value = scipy.stats.pearsonr(noabx_sample_res_category_sum_df['DOL'],
total_counts)
print('Pearson r = {0:.2f}, p = {1:.3f}'.format(pearson_r,p_value))

yesabx_sample_res_cat_by_week, yesabx_sample_res_category_sum_df =
make_cat_res_sum_by_week_df(sample_res_category_sum, True)
# create bar graph displaying infant week and mean resistance content by category
sns.set_palette(sns.color_palette("husl", 15))
fig = yesabx_sample_res_cat_by_week.plot(kind='bar', stacked=True, width=0.75,
figsize=(8,7))
plt.ylabel('counts per million reads')
plt.title('Babies that received postweek antibiotics')
handles, labels = fig.get_legend_handles_labels()
fig.legend(handles[::-1], labels[::-1], title='Resistance Mechanisms', loc='center
left', bbox_to_anchor=(1, 0.5))
```

```
plt.show()
# calculate correlation
total_counts = yesabx_sample_res_category_sum_df.sum(axis=1)
pearson_r, p_value = scipy.stats.pearsonr(yesabx_sample_res_category_sum_df['DOL'],
total_counts)
print('Pearson r = {0:.2f}, p = {1:.3f}'.format(pearson_r,p_value))
```

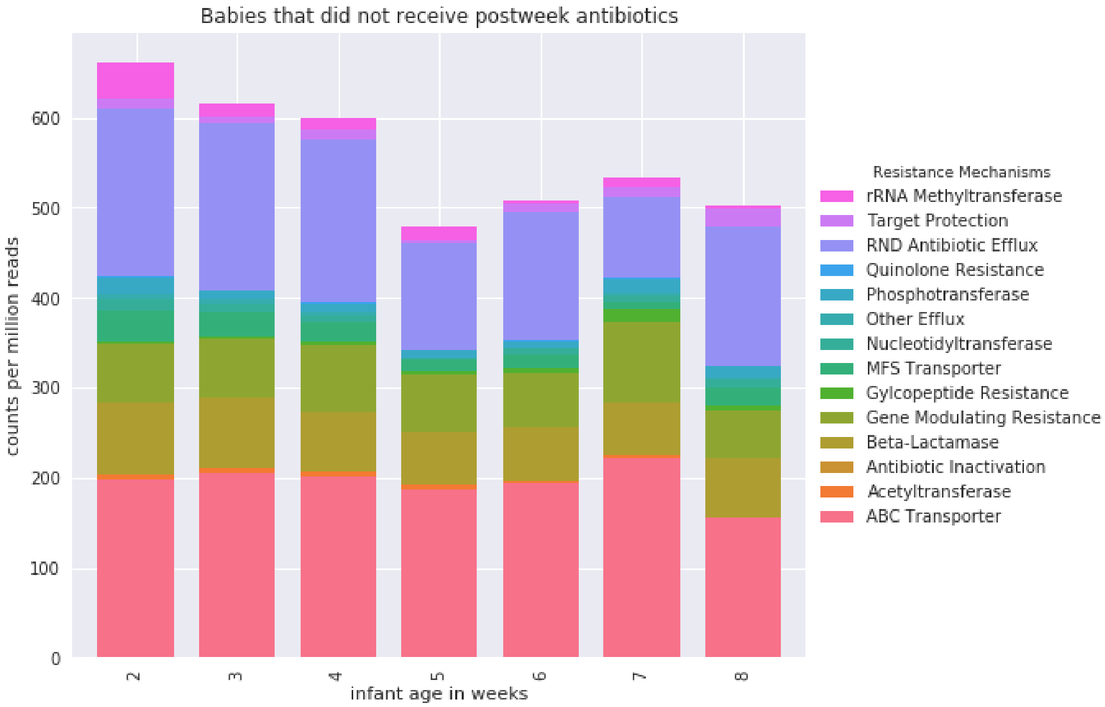


```
Pearson r = -0.12, p = 0.003
```

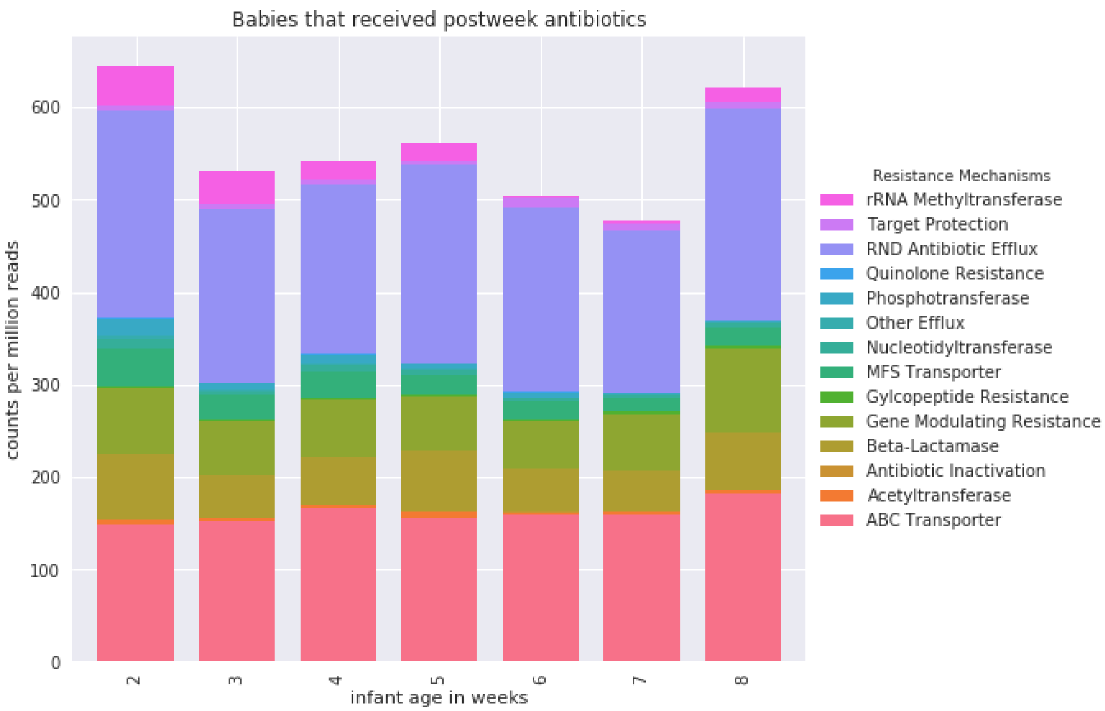


```
Pearson r = -0.07, p = 0.265
```

0.1.7 Taxonomy of the infant microbiome over time

```
In [214]: %%bash

          # unzip the folder of relative abundance data
          unzip -q relative_abundance.zip

          # create a file with a list of the rel abun csv names
          ls *_rel_abun.csv > rel_abun_filenames.txt
```

```
In [183]: def label_genus(taxonomy_df):
              """ Takes in the genome taxonomy dataframe from concoct binning
              and returns the dataframe with a new column, genus."""
              genera = [] # initialize list to contain genuses
              for l in taxonomy_df['taxonomy'].str.split():
                  genus = l[0]
                  genera.append(genus) # add the first word of the concoct taxonomy classification
              fixed_genera = [] # Some start with brackets and need to be fixed
              for genus in genera:
                  if genus[0] == '[' and genus[-1] == ']':
                      genus_fixed = genus[1:-1]
                  elif genus[0] == '[':
                      genus_fixed = genus[1:]
                  elif genus[-1] == "[":
                      genus_fixed = genus[:-1]
                  else:
                      genus_fixed = genus
                  fixed_genera.append(genus_fixed)
              taxonomy_df['genus'] = fixed_genera # add fixed genera as genus column in dataframe
              taxonomy_df = genome_taxonomy[['genome','taxonomy','genus']] # subset the dataframe
```

```
to columns of interest
    return(taxonomy_df)

def create_list_of_dfs(file):
    """Takes in a filename of a file containing a list of csvs,
    converts these csvs to dataframe, store them in a list and
    return the list."""
    list_of_dfs = []
    f = open(file)
    for line in f:
        name = line.split('\n')[0]
        data = pd.read_csv('relative_abundance_csvs/'+name)
        list_of_dfs.append(data)
    f.close()
    return(list_of_dfs)

def merge_on_genome(list_of_dfs, df_to_merge):
    """Takes in a list of dataframes and another dataframe (df_to_merge),
    adjusts the genome column
    so that the names accurately reflect the name of the fasta file,
    and merges each dataframe in the list with the df_to_merge."""
    for df in list_of_dfs:
        genome_names = []
        for genome in df['genome']:
            genome_name = genome+'.fa'
            genome_names.append(genome_name)
        df['genome'] = genome_names
    list_of_merged_dfs = []
    for df in rel_abun_dfs:
        new_df = df.merge(df_to_merge, on='genome')
        list_of_merged_dfs.append(new_df)
    return(list_of_merged_dfs)

def sum_genome_groups(list_of_dfs, class_level):
    """Take in a list of dataframs, and based on a classification
    listed in a classification column in each dataframe, sum the values
    for each member of each class to come up with values for the group
    as a whole. Return one concatenated dataframe."""
    rel_abun_dfs_of_interest = []
    for df in list_of_dfs:
        abun_cols = [col for col in df.columns if 'abun' in col]
        df2 = df.filter(items=abun_cols)
        df2[class_level] = df[class_level]
        cdif_abun_df = df2.groupby(class_level).sum()
        rel_abun_dfs_of_interest.append(cdif_abun_df)
    all_samples_df = pd.concat(rel_abun_dfs_of_interest, axis=1)
    all_samples_df = all_samples_df.fillna(0)
    new_col_names = []
    for col_name in all_samples_df.columns:
        sample = "_".join(col_name.split("_")[:-2])
        new_col_names.append(sample)
    all_samples_df.columns = new_col_names
    return(all_samples_df)

def calculate_genus_abundance(abun_df):
    abun_df_t = abun_df.transpose()
    DOLs = []
    for sample_split in abun_df_t.index.str.split("_"):
        DOL = sample_split[-1][:3]
        DOLs.append(int(DOL))
    abun_df_t['DOL'] = DOLs
    abun_df_t['week'] = abun_df_t.apply(lambda row: label_week(row),axis=1)
    genus_abundances = abun_df_t[abun_df_t['week'] != '9+']
    genus_abundances_by_week = genus_abundances.groupby('week').mean()
    genus_abundances_by_week = genus_abundances_by_week.drop('DOL',axis=1)
    return(genus_abundances_by_week)

genome_taxonomy_with_genus = label_genus(genome_taxonomy)
```

```
rel_abun_dfs = create_list_of_dfs("rel_abun_filenames.txt")
merged_rel_abun_dfs = merge_on_genome(rel_abun_dfs, genome_taxonomy_with_genus)
all_samples_abun_df = sum_genome_groups(merged_rel_abun_dfs, 'genus')
yes_abx_samples = metadata_ordered.loc[metadata_ordered['postweek_ab']==True]['sample']
no_abx_samples = metadata_ordered.loc[metadata_ordered['postweek_ab']==False]['sample']
yes_abx_abun_df = all_samples_abun_df[yes_abx_samples]
no_abx_abun_df = all_samples_abun_df[no_abx_samples]

genus_abundances_by_week_yesabx = calculate_genus_abundance(yes_abx_abun_df)
genus_abundances_by_week_noabx = calculate_genus_abundance(no_abx_abun_df)

freq_occuring_genera = []
for week in range(2,9):
    top_genera_yes_abx = list(genus_abundances_by_week_yesabx.sort_values(by=week,axis=1
,ascending=False).columns[:7])
    top_genera_no_abx = list(genus_abundances_by_week_noabx.sort_values(by=week,axis=1,a
scending=False).columns[:7])
    freq_occuring_genera.extend(top_genera_no_abx)
    freq_occuring_genera.extend(top_genera_yes_abx)
freq_occuring_genera = list(set(freq_occuring_genera))
fog_copy = freq_occuring_genera

genus_abundances_by_week_yesabx['other'] = 1 -
genus_abundances_by_week_yesabx[freq_occuring_genera].sum(axis=1)
fog_copy.extend(['other'])
genus_abundances_by_week_yesabx_toplot =
genus_abundances_by_week_yesabx[freq_occuring_genera]
sns.set_palette(sns.color_palette("hls", 18))
fig = genus_abundances_by_week_yesabx_toplot.plot(kind='bar', stacked=True, width=0.75)
handles, labels = fig.get_legend_handles_labels()
fig.legend(handles[::-1], labels[::-1], title='Genus', loc='center left',
bbox_to_anchor=(1, 0.5))
plt.ylim(0,1)
plt.ylabel('Relative abundance')
plt.xlabel('infant age in weeks')
plt.title('Infants that received postweek antibiotics')

genus_abundances_by_week_noabx['other'] = 1 -
genus_abundances_by_week_noabx[freq_occuring_genera[:-1]].sum(axis=1)
genus_abundances_by_week_noabx_toplot =
genus_abundances_by_week_noabx[freq_occuring_genera]
fig = genus_abundances_by_week_noabx_toplot.plot(kind='bar', stacked=True, width=0.75)
handles, labels = fig.get_legend_handles_labels()
fig.legend(handles[::-1], labels[::-1], title='Genus', loc='center left',
bbox_to_anchor=(1, 0.5))
plt.title('Infants that did not receive postweek antibiotics')
plt.ylabel('Relative abundance')
plt.xlabel('infant age in weeks')

plt.show()
```

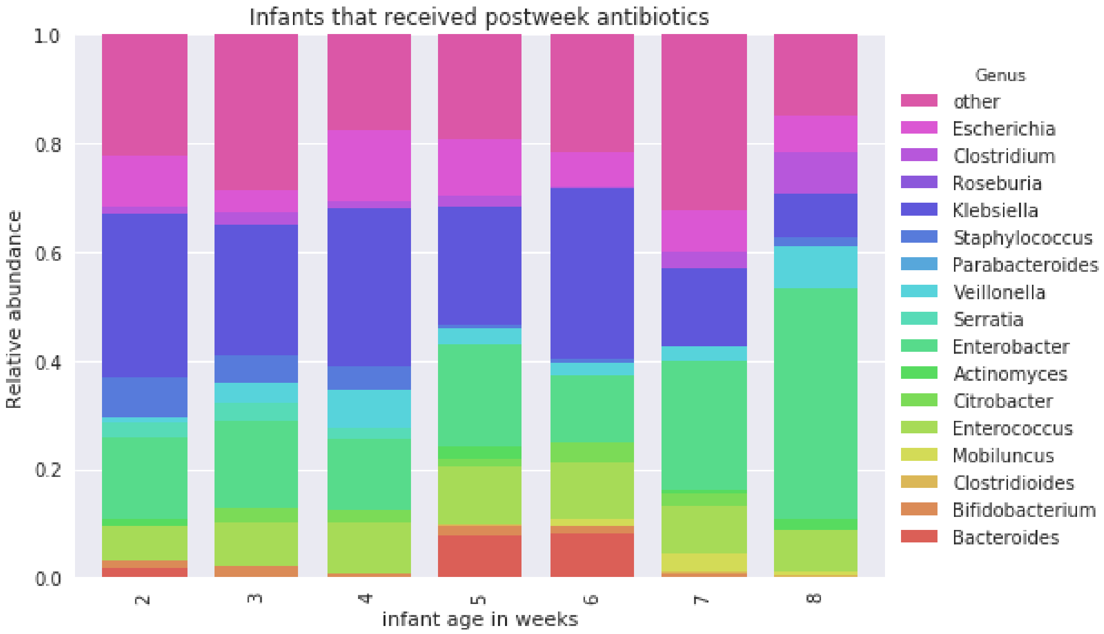

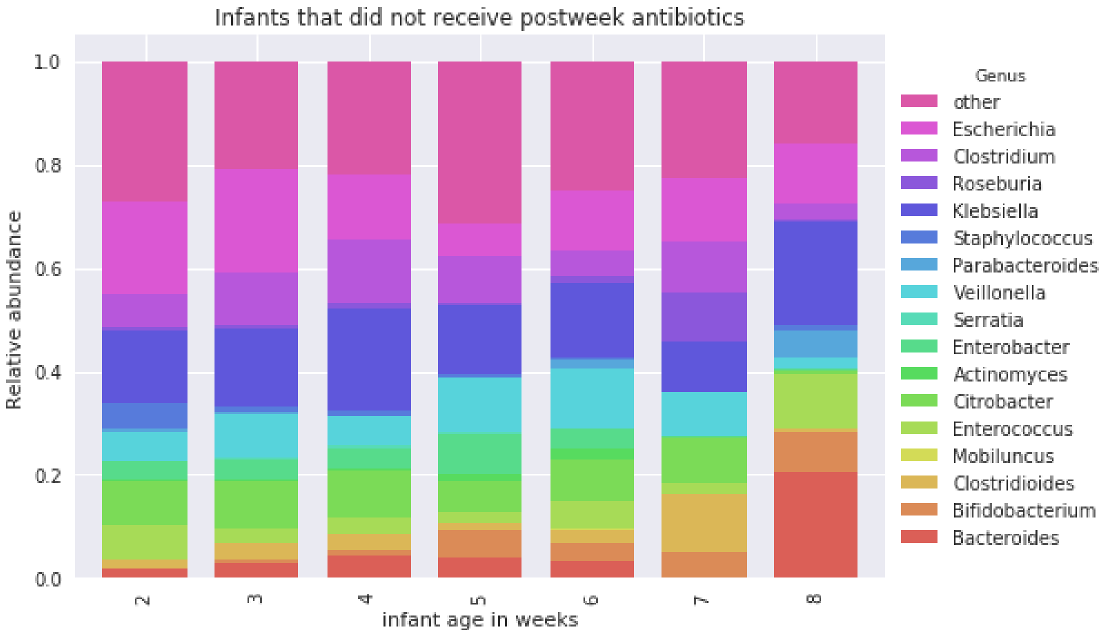

0.1.8 PERMANOVA to evaluate impact of clinical factors on resistome

To determine which factors have a significant effect on the distribution of resistance genes in the infant gut microbiome, cross-sectional PERMANOVAs were performed at discrete time points (2, 4, and 6 weeks of age) to overcome the bias of repeated measures in longitudinal sampling. The

PERMANOVA partitions the variance associated with individual factors, allowing us to determine each factor's effect on the resistome.

In [173]:
```
def create_master_data_df(res_gene_df, metadata_df):
    """Using the sample resistance gene summaries and the metadata dataframe,
    generate one dataframe that contains all the data & metadata, which can be later
broken
    up for the cross-sectional PERMANOVAs."""
    res_gene_df['sample'] = res_gene_df.index
    # merge metadata with resistance gene data
    res_gene_and_metadata = metadata_ordered.merge(res_gene_df, on='sample')
    def label_antibiotics(row):
        """Label True if the infant is receiving antibiotics
        on the day the sample is taken, Label False if it is not
        receiving antibiotics."""
        if row['antibiotics'] == '[]':
            return(False)
        else:
            return(True)
    res_gene_and_metadata['received_breastmilk'] = res_gene_and_metadata.apply(lambda
row: \
label_breastmilk(row),axis=1)
    res_gene_and_metadata['received_antibiotics'] = res_gene_and_metadata.apply(lambda
row: \
label_antibiotics(row),axis=1)
    return(res_gene_and_metadata)

def find_first_sample(week_df):
    """Return the name of the sample that is the first
    sample from each infant for a particular week"""
    seen_infant = []
    first_samples = []
    i = 0
    for infant in week_df.infant:
        if infant not in seen_infant:
            seen_infant.append(infant)
            first_samples.append(week_df.ix[i]['sample'])
            i = i + 1
        else:
            i = i + 1
    return(first_samples)

def create_cross_section_dfs(week_df):
    """To avoid having the sample infant appear in multiple samples in the same
PERMANOVA,
    select only one sample from each infant to use."""
    first_samples = find_first_sample(week_df)
    week_df_for_permanova = week_df.loc[week_df['sample'].isin(first_samples)]
    return(week_df_for_permanova)

def make_csvs_for_adonis(week_string, selected_week_df):
    """Intake a string indicating the week, and the week's dataframe with first samples.
    Save each week's data and metadata as csv files, for subsequent upload into R,
    so the PERMANOVA can be performed using the adonis2 function in the R package
vegan.)"""
    res_gene_df = selected_week_df.iloc[:, 30:200]
    metadata_df = selected_week_df[['received_breastmilk','received_antibiotics',
                                    'birth_mode','gender','maternal_ab']]
    #Save as csv files for transfer to R for PERMANOVA
    res_gene_df.to_csv(week_string+'_res_gene.csv')
    metadata_df.to_csv(week_string+'_metadata.csv')

def bonferroni_correction(p_value, num_tests):
    """Perform bonferonni corrections to adjust for multiple testing when all p-values
are not yet
    available but the number of tests is known."""
    corrected_p = p_value * float(num_tests) # Bonferonni corrections multiply the value
by the number of tests
```

```
                                              # performed (in the same family of
hypotheses)
    if corrected_p > 1:
        corrected_p = 1.0 # corrected p-values above 1 should be reduced to 1
    return(corrected_p)

def make_p_table(week_string, csv):
    """Intake PERMANOVA result csv and a string indicating the week. Bonferonni adjust
p_values, since multiple
    PERMANOVA tests were performed. Return a plotly table."""
    # load csv from R into python pandas dataframe
    result_df = pd.read_csv(csv)
    # give un-abbreviated column names
    result_df = result_df.rename(columns={'Df':'Degrees of freedom', 'SumOfSqs':'Sum of
Squares', \
                                          'F':'F-statistic', 'Pr(>F)':"p value"})
    # since three PERMANOVAs are performed, Bonferonni adjust the p-values for multiple
testing
    corrected_ps = []
    for p in result_df['p value']:
        corrected_ps.append(bonferroni_correction(p, 3))
    result_df['corrected p value'] = np.round(corrected_ps, decimals=3)
    # ensure all values in df are rounded to 3 decimal places
    for column_name in result_df.columns:
        result_df[column_name] = np.round(result_df[column_name], decimals=3)
    # create and return plotly table
    result_df = result_df.fillna(value='')
    return(result_df)

master_res_gene_metadata = create_master_data_df(sample_res_gene_ordered,
metadata_ordered)

# Create dataframes for weeks 2, 4, and 6, from which to draw samples for the cross-
sectional PERMANOVAs
res_gene_and_metadata_week2 = master_res_gene_metadata.loc \
[master_res_gene_metadata['DOL'].between(8, 14) == True].reset_index()
res_gene_and_metadata_week4 = master_res_gene_metadata.loc \
[master_res_gene_metadata['DOL'].between(22, 28) == True].reset_index()
res_gene_and_metadata_week6 = master_res_gene_metadata.loc \
[master_res_gene_metadata['DOL'].between(36, 42) == True].reset_index()

# perform PERMANOVAs
weeks_and_dfs = [('week2', res_gene_and_metadata_week2), ('week4',
res_gene_and_metadata_week4),
                 ('week6', res_gene_and_metadata_week6)]
permanova_dfs = []
for week, df in weeks_and_dfs:
    permanova_df = create_cross_section_dfs(df)
    permanova_dfs.append(permanova_df)
    make_csvs_for_adonis(week, permanova_df)
```

In [218]:

```
%R library(vegan)

%R sample_resistance_gene_summaries = read.table('week2_res_gene.csv', header=TRUE,
sep=",")
%R rownames(sample_resistance_gene_summaries) <- sample_resistance_gene_summaries$index
# Load metadata and generate labels for factors
%R metadata_for_permanova = read.table('week2_metadata.csv', header=TRUE, sep=",")
%R birth_mode <- metadata_for_permanova$birth_mode
%R gender <- metadata_for_permanova$gender
%R maternal_antibiotics <- metadata_for_permanova$maternal_ab
%R infant_antibiotics <- metadata_for_permanova$received_antibiotics
%R received_breastmilk <- metadata_for_permanova$received_breastmilk
# Perform marginal PERMANOVA using Bray-Curtis dissimilarty metric
%R permanova_result <- adonis2(formula = sample_resistance_gene_summaries ~ \
                              received_breastmilk + infant_antibiotics + birth_mode + \
                              gender + maternal_antibiotics, data =
sample_resistance_gene_summaries, \
```

```
                               method = 'bray', by="margin", parallel = 9, permutations
= 9999)
%R write.table(permanova_result, file="week2_permanova_result.csv", sep=",")

%R sample_resistance_gene_summaries = read.table('week4_res_gene.csv', header=TRUE,
sep=",")
%R rownames(sample_resistance_gene_summaries) <- sample_resistance_gene_summaries$index
# Load metadata and generate labels for factors
%R metadata_for_permanova = read.table('week4_metadata.csv', header=TRUE, sep=",")
%R birth_mode <- metadata_for_permanova$birth_mode
%R gender <- metadata_for_permanova$gender
%R maternal_antibiotics <- metadata_for_permanova$maternal_ab
%R infant_antibiotics <- metadata_for_permanova$received_antibiotics
%R received_breastmilk <- metadata_for_permanova$received_breastmilk
# Perform marginal PERMANOVA using Bray-Curtis dissimilarty metric
%R permanova_result <- adonis2(formula = sample_resistance_gene_summaries ~ \
                               received_breastmilk + infant_antibiotics + birth_mode + \
                               gender + maternal_antibiotics, data =
sample_resistance_gene_summaries, \
                               method = 'bray', by="margin", parallel = 9, permutations
= 9999)
%R write.table(permanova_result, file="week4_permanova_result.csv", sep=",")

%R sample_resistance_gene_summaries = read.table('week6_res_gene.csv', header=TRUE,
sep=",")
%R rownames(sample_resistance_gene_summaries) <- sample_resistance_gene_summaries$index
# Load metadata and generate labels for factors
%R metadata_for_permanova = read.table('week6_metadata.csv', header=TRUE, sep=",")
%R birth_mode <- metadata_for_permanova$birth_mode
%R gender <- metadata_for_permanova$gender
%R maternal_antibiotics <- metadata_for_permanova$maternal_ab
%R infant_antibiotics <- metadata_for_permanova$received_antibiotics
%R received_breastmilk <- metadata_for_permanova$received_breastmilk
# Perform marginal PERMANOVA using Bray-Curtis dissimilarty metric
%R permanova_result <- adonis2(formula = sample_resistance_gene_summaries ~ \
                               received_breastmilk + infant_antibiotics + birth_mode + \
                               gender + maternal_antibiotics, data =
sample_resistance_gene_summaries, \
                               method = 'bray', by="margin", parallel = 9, permutations
= 9999)
%R write.table(permanova_result, file="week6_permanova_result.csv", sep=",")
```

In [175]:
```
list_of_cvs = [("week2_permanova_result.csv",'Week 2'),
               ("week4_permanova_result.csv",'Week 4'),
               ("week6_permanova_result.csv",'Week 6')]

permanova_result_tables = []
for csv, week in list_of_cvs:
    table = make_p_table(week, csv)
    permanova_result_tables.append(table)
```

In [176]:
```
# Due to the randomness of permutations, p values are not identical to those appearing
# in the supplementary material of the publication

permanova_result_tables[0] # Week 2 PERMANOVA results
```

Out[176]:

	Degrees of freedom	Sum of Squares	F-statistic	p value	\
received_breastmilk	1	0.316	2.233	0.052	
infant_antibiotics	1	0.396	2.794	0.025	
birth_mode	1	0.188	1.324	0.208	
gender	1	0.097	0.684	0.627	
maternal_antibiotics	1	0.285	2.009	0.07	

```
Residual                                83          11.757

                     corrected p value
received_breastmilk              0.157
infant_antibiotics               0.074
birth_mode                       0.625
gender                               1
maternal_antibiotics             0.211
Residual
```

In [177]: permanova_result_tables[1] # Week 4 PERMANOVA results

```
Out[177]:                       Degrees of freedom  Sum of Squares  F-statistic  p value  \
          received_breastmilk                    1           0.535        4.202    0.004
          infant_antibiotics                     1           0.082        0.647    0.604
          birth_mode                             1           0.243        1.908    0.075
          gender                                 1           0.117        0.918    0.432
          maternal_antibiotics                   1           0.213        1.676    0.113
          Residual                              64           8.147

                               corrected p value
          received_breastmilk               0.01
          infant_antibiotics                   1
          birth_mode                       0.224
          gender                               1
          maternal_antibiotics             0.339
          Residual
```

In [178]: permanova_result_tables[2] # Week 6 PERMANOVA results

```
Out[178]:                       Degrees of freedom  Sum of Squares  F-statistic  p value  \
          received_breastmilk                    1           0.462        5.005    0.004
          infant_antibiotics                     1           0.099        1.077    0.317
          birth_mode                             1           0.087        0.941    0.413
          gender                                 1           0.160        1.735    0.132
          maternal_antibiotics                   1           0.050        0.542    0.739
          Residual                              25           2.307

                               corrected p value
          received_breastmilk              0.013
          infant_antibiotics               0.952
          birth_mode                           1
          gender                           0.395
          maternal_antibiotics                 1
          Residual
```

0.1.9 Random forest model and nonparametric test to identify genes influenced by feeding

The above results from the PERMANOVA test reveal that in weeks 4 and 6, the feeding type (i.e. whether or not the infant received breastmilk) had a significant effect on the infant's gut resistome

(p < 0.01). To identify the genes contributing to the differences of breast-fed and formula-fed infant gut resistomes, a random forest model was built, which classified resistomes as belonging to either a breast-fed or formula-fed infant, and feature importance scores were assessed. By selecting a few features for hypothesis testing, the number of comparisons that needed to be made was succesfully reduced from 170 to 4. Mann-Whitney U tests led to the identification of one gene significantly associated with the resistomes of formula-fed infants.

In [187]:
```
def model_and_test(week_res_gene_df):
    """Takes in a dataframe that combines resistance gene summaries with feeding
metadata, and
    trains a random forest model to classify each sample based on feeding type. Returns
the most
    important features of the model based on gini importance scores, along with the
results of a
    Mann-Whitney U test on these features."""
    # select resistance gene summaries of breast-fed and formula-fed babies
    breast_formula_connected_df = week_res_gene_df.loc[week_res_gene_df['feeding'] !=
'Combination']
    resistance_gene_summaries_df = breast_formula_connected_df.ix[:,31:201]
    res_gene_summaries = resistance_gene_summaries_df.values
    res_gene_summaries_scaled = preprocessing.scale(res_gene_summaries)
    output_array = np.asarray(breast_formula_connected_df['feeding'])
    # Random forest classification
    clf = RandomForestClassifier(n_estimators=10, random_state=1) # the classifier
    clf = clf.fit(res_gene_summaries_scaled, output_array) # fit model
    importances = clf.feature_importances_ # extract feature importances
    indices = np.argsort(importances)[::-1]
    resfam_ids = sample_res_gene_ordered.columns[:-1]
    # determine which Resfams were important features
    important_features = []
    resfam_to_score = {}
    i = 0
    for score in importances:
        # select resistance genes with feature importances above 0.07
        if score > 0.07:
            important_features.append(resfam_ids[i])
            resfam_to_score[resfam_ids[i]] = score
        i = i + 1
    # nonparametric testing of important features (> 0.07) to determine group
differences
    breast_df = breast_formula_connected_df.loc[breast_formula_connected_df['feeding']
== 'Breast']
    formula_df = breast_formula_connected_df.loc[breast_formula_connected_df['feeding']
== 'Formula']
    resfam_to_TestResult = {}
    for resfam in important_features:
        breast = breast_df[resfam]
        formula = formula_df[resfam]
        # compare groups with Mann-Whitney U-test
        mann_whitney_u_statistic, p_value = scipy.stats.mannwhitneyu(breast, formula)
        resfam_to_TestResult[resfam] = (mann_whitney_u_statistic, p_value)
    # build dataframes of feature_to_score and feature_to_test_result
    resfam_to_score_df = pd.DataFrame.from_dict(resfam_to_score, orient='index')
    resfam_to_score_df = resfam_to_score_df.rename(columns={0: "Feature importance
score"})
    resfam_to_TestResult_df = pd.DataFrame.from_dict(resfam_to_TestResult,
orient='index')
    resfam_to_TestResult_df = resfam_to_TestResult_df.rename(columns={0: "Mann-Whitney U
Statistic", 1: "p value"})
    result_df = resfam_to_score_df.join(resfam_to_TestResult_df, how='outer')
    return(result_df)

weeks_of_interest = [permanova_dfs[1], permanova_dfs[2]] # weeks that showed significant
result in PERMANOVA
week_result_dfs = []
for week in weeks_of_interest:
```

```
    df = model_and_test(week)
    week_result_dfs.append(df)

cat_df = pd.concat(week_result_dfs)
p_values = cat_df['p value']
# adjust p-values for multiple testing (four tests performed after feature selection
through importance scores)
correct = statsmodels.sandbox.stats.multicomp.multipletests(p_values,
method='bonferroni')
cat_df['corrected p value'] = correct[1]
cat_df = cat_df.round(decimals=3)
cat_df['Resfam ID'] = cat_df.index
# convert accession to descriptions
resfams_descriptions = resfams_metadata[['Resfam ID', 'Description']]
final_df = resfams_descriptions.merge(cat_df, on='Resfam ID')
final_df = final_df.drop('Resfam ID', axis=1)
final_df = final_df.rename(columns={'Description':'Resfam'})
# give abbreviated names to the identified antibiotic resistance genes
shortened_names = ['ANT6','Class D beta-lactamases','mexX', 'soxR mutant']
final_df['Resfam'] = shortened_names
final_df
```

Out[187]:

```
                    Resfam  Feature importance score  \
0                     ANT6                     0.071
1  Class D beta-lactamases                     0.089
2                     mexX                     0.098
3              soxR mutant                     0.071

   Mann-Whitney U Statistic  p value  corrected p value
0                      17.0    0.327              1.000
1                      66.0    0.008              0.031
2                      11.0    0.106              0.426
3                      10.0    0.081              0.324
```

In [188]:

```
def resize_plot_text(labelsize, titlesize):
    """Resize particular aspects of a matplotlib plot
    based on an input label size and title size."""
    ax = plt.subplot()
    for ticklabel in (ax.get_xticklabels()):
        ticklabel.set_fontsize(labelsize)
    for ticklabel in (ax.get_yticklabels()):
        ticklabel.set_fontsize(labelsize)
    ax.xaxis.get_label().set_fontsize(labelsize)
    ax.yaxis.get_label().set_fontsize(labelsize)
    ax.title.set_fontsize(titlesize)

# Draw boxplot to visualize difference between breast-fed and formula-fed infants for
class D betalactamase
week4_res_gene = permanova_dfs[1]
week_4_breast = week4_res_gene.loc[week4_res_gene['feeding']=='Breast']
week_4_formula = week4_res_gene.loc[week4_res_gene['feeding']=='Formula']
corrected_breast = week_4_breast['RF0056'].reset_index()
corrected_formula = week_4_formula['RF0056'].reset_index()
plt.figure(figsize=(5,3))
bp0 = plt.boxplot([corrected_breast['RF0056'], corrected_formula['RF0056']],
                  labels=['breastmilk','formula'], patch_artist=True, showfliers=False )
bp0['boxes'][0].set(color='blue', linewidth=2)
bp0['boxes'][1].set(color='red', linewidth=2)
bp0['whiskers'][3].set(color='red', linewidth=2)
for median in bp0['medians']:
    median.set(color='black', linewidth=0.8)
plt.gca().xaxis.grid(False)
plt.ylim(-0.2,8.5)
```

```
plt.ylabel('counts per million reads')
plt.title('Class D betalactamase in gut resistome at age 1 month')
resize_plot_text(10, 10)
plt.show()
mann_whitney_u_stat = str(final_df.ix[1].values[2]) # extract statistic from mann
whitney test result
corrected_p = str(final_df.ix[1].values[4]) # extract boferonni corrected p-value
print('Mann Whitney U = '+mann_whitney_u_stat+', p = '+corrected_p)
```

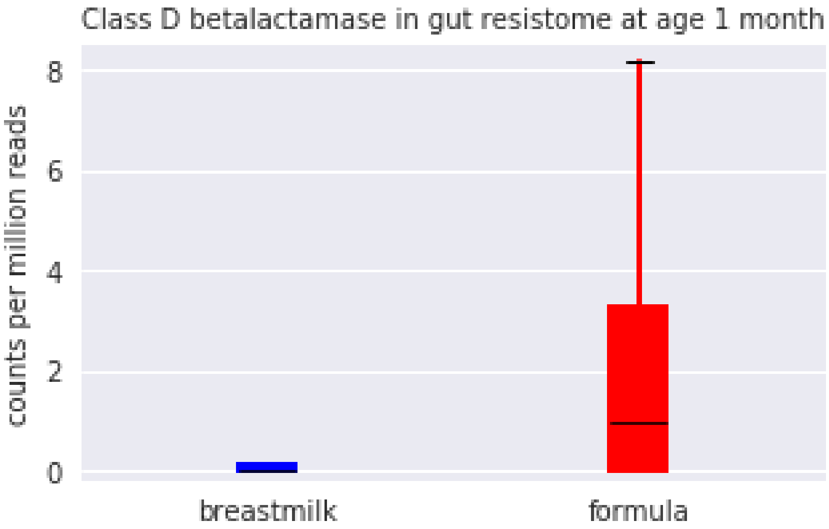


```
Mann Whitney U = 66.0, p = 0.031
```

In [189]:
```
# Determine which species of bacteria is the most frequent carrier of Class D beta-
lactamase

# merge genome res summaries with taxonomy
genome_resistance_gene_summaries['genome'] = genome_resistance_gene_summaries.index
genome_resistance_gene_summaries_tax =
genome_resistance_gene_summaries.merge(genome_taxonomy, on='genome')
# Identify the genomes that have RF0056 (Class D Betalactamase)
genomes_with_RF0056_df =
genome_resistance_gene_summaries_tax.loc[genome_resistance_gene_summaries_tax['RF0056']
> 0]
# 107 out of 1428 genomes have RF0056
# Visualize which species are the most common carriers of this gene
species_RF0056 = genomes_with_RF0056_df['taxonomy']
# count the number of each taxonomy that contains RF0056
species_RF0056_freq_dict = collections.Counter(species_RF0056)
# order the dictionary by which species are the most frequent carriers
species_most_common = species_RF0056_freq_dict.most_common()
# the total number of genomes carrying the RF0056 gene
total = sum(species_RF0056_freq_dict.values())
# the top three species and all the others grouped into "other"
minus_top_three = total -
(species_most_common[0][1]+species_most_common[1][1]+species_most_common[2][1])
# create a donut chart that displays the breakdown
labels = species_most_common[0][0], species_most_common[1][0],
species_most_common[2][0], 'other'
sizes = [species_most_common[0][1]/total, species_most_common[1][1]/total, \
         species_most_common[2][1]/total, minus_top_three/total]
```

```
colors = ['yellowgreen', 'gold', 'lightskyblue', 'lightcoral']
explode = (0, 0, 0, 0)  # explode a slice if required
plt.figure(figsize=(4.3,4.3))
plt.pie(sizes, explode=explode, labels=labels, colors=colors,
        autopct='%1.1f%%', shadow=True)
centre_circle = plt.Circle((0,0),0.75,color='black', fc='white',linewidth=1.25)
fig = plt.gcf()
fig.gca().add_artist(centre_circle)
plt.axis('equal')
plt.title('Genomes harboring Class D betalactamase')
plt.tight_layout()
plt.show()
```

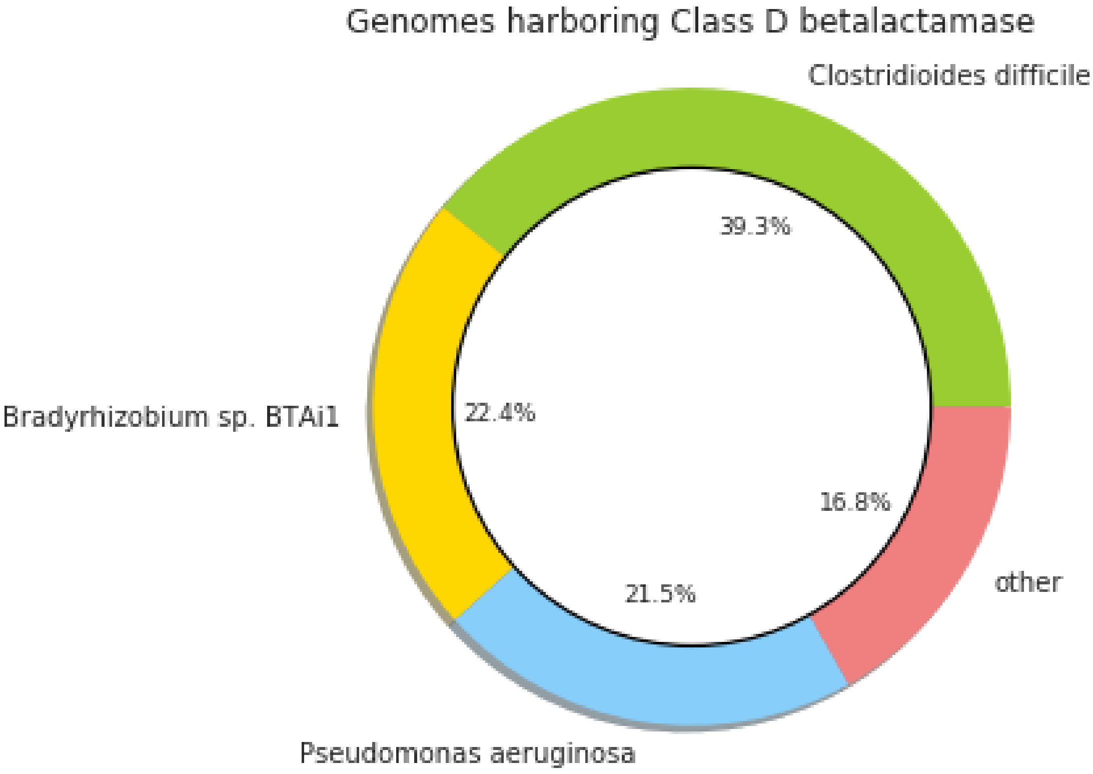

0.1.10 *Clostridium difficile* with Class D betalactamase and the connection to formula-fed infants

In the visualizations above, it is shown that Class D betalactamase is enriched in formula-fed infants and the species that most frequently harbors this gene is *Clostridiodes difficile*, also known as *Clostridium difficile*. Phylogenetic analysis (through alignment of RPS3 gene with MUSCLE and generation of maximum-liklihood phylogenetic tree with raXml) revealed that the *C. difficile* harboring the Class D betalactamase formed a clonal group. The phylogenetic tree can be built using the alignment fasta file in this github repository. Next, we decipher the abundance dynamics of *C. difficile*. The analysis shows that *C. difficile* with Class D betalactamase is consistently more abundant in formula-fed infants than *C. difficile* without this gene. The presence of one gene that may be involved in carbohydrate transport is perfectly correlated with the presence of Class D betalactamase.

```
In [190]: def select_genome(df, species):
              """Select genomes of a species of interest from a dataframe
              with a taxonomy column, return the selected part of the dataframe
              and list of these genomes."""
              species_df = df.loc[df['taxonomy'] == species]
              list_of_genomes = species_df['genome'].tolist()
              return(species_df, list_of_genomes)

          def abundance_through_time_by_feeding(groups_to_test, week_dfs, all_sample_df,
          metadata_df):
              """Returns a list of lists of the groups' relative abundance through the
              weeks specified by the week dfs, along with the list of lists of standard
              errors for each of those datapoints, grouped by feeding type."""
              group_to_results = {}
              group_to_error = {}
              for group in groups_to_test:
                  formula_abundance_datapoints = []
                  all_y_error_formula = []
                  breast_abundance_datapoints = []
                  all_y_error_breast = []
                  for df in week_dfs:
                      week_X_samples = find_first_sample(df)
                      week_X_cols = [col for col in all_sample_df.columns if col[:12] in
          week_X_samples]
                      week_X_species_matrix = all_sample_df.filter(items=week_X_cols)
                      week_X_data_matrix1 = week_X_species_matrix.transpose()
                      week_X_data_matrix2 =
          week_X_data_matrix1.loc[~(week_X_data_matrix1==0).all(axis=1)]
                      week_X_sample_list = []
                      for entry in week_X_data_matrix2.index:
                          week_X_sample_list.append(entry[:12])
                      week_X_data_matrix2['sample'] = week_X_sample_list
                      week_X_data_matrix_feeding = week_X_data_matrix2.merge(metadata_df,
          on='sample', how='inner')
                      week_X_data_matrix_feeding.index = week_X_data_matrix2['sample']
                      week_X_data_matrix_breast = week_X_data_matrix_feeding.loc \
          [week_X_data_matrix_feeding['received_breastmilk']==True]
                      week_X_data_matrix_formula = week_X_data_matrix_feeding.loc \
          [week_X_data_matrix_feeding['received_breastmilk'] != True]
                      mean_abun_for_formula = week_X_data_matrix_formula[group].mean()
                      formula_abundance_datapoints.append(mean_abun_for_formula)
                      mean_abun_for_breast = week_X_data_matrix_breast[group].mean()
                      breast_abundance_datapoints.append(mean_abun_for_breast)
                      y_error_formula = scipy.stats.sem(week_X_data_matrix_formula[group], ddof=0)
                      all_y_error_formula.append(y_error_formula)
                      y_error_breast = scipy.stats.sem(week_X_data_matrix_breast[group], ddof=0)
                      all_y_error_breast.append(y_error_breast)
                  group_to_results[group] = [formula_abundance_datapoints,
          breast_abundance_datapoints]
                  group_to_error[group] = [all_y_error_formula, all_y_error_breast]
              return(group_to_results, group_to_error)


          rel_abun_dfs = create_list_of_dfs('rel_abun_filenames.txt')

          genomes_with_RF0056_df = genome_resistance_gene_summaries_tax.loc \
                                  [genome_resistance_gene_summaries_tax['RF0056'] > 0]
          c_dif_with_RF0056_df, c_dif_with_RF0056 = select_genome(genomes_with_RF0056_df,
          'Clostridioides difficile')

          genomes_lacking_RF0056_df = genome_resistance_gene_summaries_tax.loc \
                                     [genome_resistance_gene_summaries_tax['RF0056'] == 0]
          c_dif_lacking_RF0056_df, c_dif_lacking_RF0056 = select_genome(genomes_lacking_RF0056_df,
          'Clostridioides difficile')

          # For each rel_abun_df, identify and label C. difficile with and without RF0056
          for df in rel_abun_dfs:
              classification_list = []
```

```
    for genome in df['genome']:
        if genome+'.fa' in c_dif_with_RF0056:
            classification_list.append('c_dif_with_RF0056')
        elif genome+'.fa' in c_dif_lacking_RF0056:
            classification_list.append('c_dif_lacking_RF0056')
        else:
            classification_list.append('neither')
    df['classification'] = classification_list

all_samples_cdif_abun_df = sum_genome_groups(rel_abun_dfs, 'classification')

# Data frames for weeks 2, 4, and 6 have already been created, do the same for weeks 3
and 5
res_gene_and_metadata_week3 = master_res_gene_metadata.loc \
                            [master_res_gene_metadata['DOL'].between(15, 21) ==
True].reset_index()
res_gene_and_metadata_week5 = master_res_gene_metadata.loc \
                            [master_res_gene_metadata['DOL'].between(29, 35) ==
True].reset_index()
week_dfs = [res_gene_and_metadata_week2, res_gene_and_metadata_week3,
res_gene_and_metadata_week4,
           res_gene_and_metadata_week5, res_gene_and_metadata_week6]
groups_to_test = ['c_dif_with_RF0056', 'c_dif_lacking_RF0056']
# calculate relative abundance datapoints and standard error for error bars for each
datapoint
datapoints_dict, error_dict = abundance_through_time_by_feeding(groups_to_test,
week_dfs, all_samples_cdif_abun_df,
                                                               master_res_gene_metadata)

# Generate figure to show the abundance of C. difficile groups (with and without RF0056)
over time
fig = plt.figure(figsize=(8,4))
ax1 = plt.subplot(2,1,1)
plt.title("Clostridium difficile with Class D Beta-lactamase")
formula_points = datapoints_dict['c_dif_with_RF0056'][0]
breast_points = datapoints_dict['c_dif_with_RF0056'][1]
ax1.errorbar(x=np.arange(0,5), y=formula_points,
yerr=error_dict['c_dif_with_RF0056'][0], color='red')
ax1.errorbar(x=np.arange(0,5), y=breast_points, yerr=error_dict['c_dif_with_RF0056'][1],
color='blue')
formula = mpatches.Patch(color='red', label='received formula only')
breast = mpatches.Patch(color='blue', label='received breastmilk')
leg = plt.legend(handles=[formula, breast], loc='upper left', prop={'size': 10})
leg.get_frame().set_linewidth(0.0)
plt.ylabel('Relative abundance')
plt.legend()
plt.ylim(-0.01,0.13)
plt.xlim(-0.2,4.2)
ax1.set_xticklabels(["",2,3,4,5,6])
ax1.yaxis.set_ticks(np.arange(0, 0.15, 0.03))
for item in ([ax1.title, ax1.xaxis.label, ax1.yaxis.label] +
             ax1.get_xticklabels() + ax1.get_yticklabels()):
    item.set_fontsize(10)

ax2 = plt.subplot(2,1,2)
plt.title("Clostridium difficile lacking Class D Beta-lactamase")
formula_points = datapoints_dict['c_dif_lacking_RF0056'][0]
breast_points = datapoints_dict['c_dif_lacking_RF0056'][1]
ax2.errorbar(x=np.arange(0,5), y=formula_points,
yerr=error_dict['c_dif_lacking_RF0056'][0], color='red')
ax2.errorbar(x=np.arange(0,5), y=breast_points,
yerr=error_dict['c_dif_lacking_RF0056'][1], color='blue')
plt.ylabel('Relative abundance')
plt.xlabel('Infant age in weeks')
plt.legend()
plt.ylim(-0.01,0.13)
plt.xlim(-0.2,4.2)
ax2.set_xticklabels(["",2,3,4,5,6])
```

```
ax2.yaxis.set_ticks(np.arange(0, 0.15, 0.03))
for item in ([ax2.title, ax2.xaxis.label, ax2.yaxis.label] +
             ax2.get_xticklabels() + ax2.get_yticklabels()):
    item.set_fontsize(10)

plt.tight_layout()
plt.show()
```

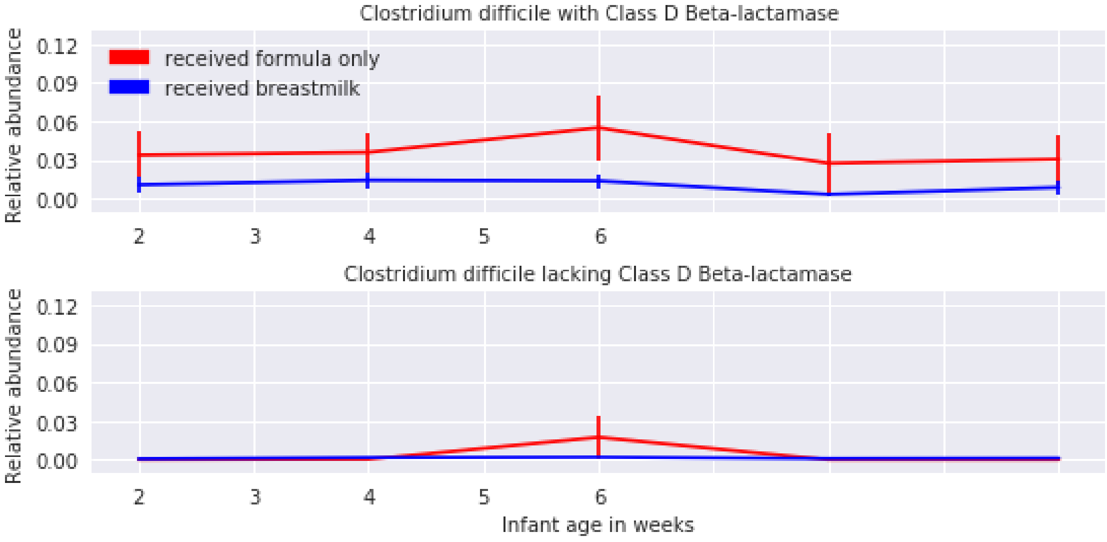

In [191]:
```
# Ensure that low relative abundance did not cause poor reconstruction of C. difficile
genomes and lead to
# lack of betalactamase gene in the genome

c_dif_lacking_RF0056_metrics = genome_metrics.loc[genome_metrics['Bin
Id'].isin(c_dif_lacking_RF0056)]
c_dif_with_RF0056_metrics = genome_metrics.loc[genome_metrics['Bin
Id'].isin(c_dif_with_RF0056)]

# Check if there is a significant different in the completeness (measured by presence of
# bacterial single copy genes) of the two C. dif groups
U_stat, p_val = scipy.stats.mannwhitneyu(c_dif_lacking_RF0056_metrics['Completeness'],
c_dif_with_RF0056_metrics['Completeness'])
print("There is no significant difference in the completeness of the two groups, p
=",p_val)

# Check if there is a sig dif in N50 between the two groups
U_stat, p_val = scipy.stats.mannwhitneyu(c_dif_lacking_RF0056_metrics['N50 (contigs)'],
c_dif_with_RF0056_metrics['N50 (contigs)'])
print("There is no significant difference in the N50 of the two groups, p =",p_val)

# Check if all the strains in one group occured in infants from the same area of the
NICU
c_dif_lacking_RF0056_infants = [] # initialize list of infants containing c dif that
lack RF0056
for genome in c_dif_lacking_RF0056:
    infant = "_".join(genome.split("_")[:2]).split("con")[1]
    c_dif_lacking_RF0056_infants.append(infant)
c_dif_with_RF0056_infants = [] # initialize list of infants containing c dif that have
RF0056
for genome in c_dif_with_RF0056:
    infant = "_".join(genome.split("_")[:2]).split("con")[1]
```

```
            c_dif_with_RF0056_infants.append(infant)

        c_dif_lacking_RF0056_infant_pod = infant_metadata.loc[infant_metadata['infant'].isin(c_d
        if_lacking_RF0056_infants)]['first_pod']
        c_dif_with_RF0056_infant_pod = infant_metadata.loc[infant_metadata['infant'].isin(c_dif_
        with_RF0056_infants)]['first_pod']
        c_dif_lacking_RF0056_infant_pod_counts =
        list(c_dif_lacking_RF0056_infant_pod.value_counts())
        c_dif_with_RF0056_infant_pod_counts = list(c_dif_with_RF0056_infant_pod.value_counts())

        obs = np.array([c_dif_lacking_RF0056_infant_pod_counts,
        c_dif_with_RF0056_infant_pod_counts])
        chi2, p, dof, expected = chi2_contingency(obs)
        print('There is no significant different in the locations of the infants with each
        strain type, p =', p)
```

```
There is no significant difference in the completeness of the two groups, p =
0.293668170217
There is no significant difference in the N50 of the two groups, p = 0.452088868376
There is no significant different in the locations of the infants with each strain
type, p = 0.867135334778
```

In [192]:
```
# To explore possible reasons why C. difficile with Class D betalactamase has a
selective advantage
# in formula-fed infants, search for metabolic pathways correlated with Class D
betalactamase

def make_accession_dict(kegg_annotation_df, resfams_metadata_df):
    """Return a dictionary that connects the accession numbers of Resfams and KEGG
modules
    (keys in the dictionary) to the full descriptions of the genes or pathways
(values)."""
    accession_to_gene_dict = {}
    for module in kegg_annotation_df.Module:
        description =
kegg_annotation_df.loc[kegg_annotation_df['Module']==module]['Description']
        pathway = description.to_string()[1:]
        accession_to_gene_dict[module] = pathway
    for accession in resfams_metadata_df['Resfam ID']:
        description = resfams_metadata_df.loc[resfams_metadata_df['Resfam
ID']==accession]['Description']
        gene = description.to_string()[1:]
        accession_to_gene_dict[accession] = gene
    return(accession_to_gene_dict)

def calculate_kegg_correlation(genome_res_df, KEGG_genome_profiles, resfam_accession):
    """Calculates pairwise Pearson correlation coefficients for a Resfam with
    all the KEGG modules, for a particular set of genomes in the input
    dataframe of genome resistance gene summaries."""
    KEGG_results_df_t = KEGG_genome_profiles.transpose()
    KEGG_results_df_t.columns = KEGG_results_df_t.ix[0]
    KEGG_results_df_t['genome'] = KEGG_results_df_t.index
    merged_df = KEGG_results_df_t.merge(genome_res_df, on='genome')
    modules_df = merged_df.ix[:,:614]
    modules_df[resfam_accession] = merged_df[resfam_accession]
    modules_df = modules_df.astype(float)
    correlation_df = modules_df.corr()
    sorted_resfam_corr = correlation_df.sort_values(by=resfam_accession,
ascending=False)[resfam_accession]
    sorted_resfam_corr_df = sorted_resfam_corr.to_frame()
    # Instead of module accession numbers, list the descriptive names of the modules
    description_replacements = []
    for accession in sorted_resfam_corr_df.index:
        description = accession_to_gene_dict[accession]
        description_replacements.append(description)
    sorted_resfam_corr_df['description'] = description_replacements
```

```
    return(sorted_resfam_corr_df)

def find_perfect_correlations(corr_df, value_of_interest):
    """Using the input correlation dataframe, identify the variable that is
    perfectly correlated with the value of interest (Pearson correlation coefficient =
1.0)."""
    perfect_corrs = corr_df.loc[corr_df[value_of_interest] == 1]
    perfect_corrs = perfect_corrs.loc[perfect_corrs.index != value_of_interest]
    return(perfect_corrs)

c_dif_genome_res_df, c_dif_genomes = select_genome(genome_resistance_gene_summaries_tax,
'Clostridioides difficile')
accession_to_gene_dict = make_accession_dict(KEGG_genome_profiles, resfams_metadata)
modules_corr_with_RF0056 = calculate_kegg_correlation(c_dif_genome_res_df,
KEGG_genome_profiles, 'RF0056')
correlated_module = find_perfect_correlations(modules_corr_with_RF0056, 'RF0056')
# the CMP-KDO biosynthesis always appears in C. difficile genomes containing Class D
betalactamase (Pearson's r = 1.0)
print(correlated_module)

# There are mutiple genes that could potentially appear in the CMP-KDO biosynthesis
module
# Determine which gene(s) from that module actually in the C. difficile genomes
KEGG_gene_annotations_c_dif =
KEGG_gene_annotations.loc[KEGG_gene_annotations['Bin'].isin(c_dif_genomes)]
M00063_in_c_dif =
KEGG_gene_annotations_c_dif.loc[KEGG_gene_annotations_c_dif['Module']=='M00063']
genes = M00063_in_c_dif['Description'].unique()

print('\n The following gene(s) in the module correlated with Class D betalactamase
occur in C. difficile: ')
for gene in genes:
    print(gene)
```

```
        RF0056                  description
M00063     1.0   1    CMP-KDO biosynthesis

 The following gene(s) in the module correlated with Class D betalactamase occur in C.
difficile:
arabinose-5-phosphate isomerase [EC:5.3.1.13]
```

In [196]:
```
# Check that Bifidobacterium is more prevalent in infants that received breastmilk
genera = []
for l in genome_taxonomy['taxonomy'].str.split():
    genus = l[0]
    genera.append(genus)
genome_taxonomy['genus'] = genera

def select_genome_genus(df, genus):
    """Select genomes of a genus of interest from a dataframe
    with a taxonomy column, return the selected part of the dataframe
    and list of these genomes."""
    genus_df = df.loc[df['genus'] == genus]
    list_of_genomes = genus_df['genome'].tolist()
    return(genus_df, list_of_genomes)

bifido_df, bifidobacterium = select_genome_genus(genome_taxonomy, 'Bifidobacterium')

# For each rel_abun_df, identify and label Bifidobacterium
for df in rel_abun_dfs:
    classification_list = []
    for genome in df['genome']:
        if genome+'.fa' in bifidobacterium:
            classification_list.append('bifidobacterium')
        else:
            classification_list.append('not')
```

```
    df['classification'] = classification_list

all_samples_bifido_abun_df = sum_genome_groups(rel_abun_dfs, 'classification')
groups_to_test = ['bifidobacterium']
# calculate relative abundance datapoints and standard error for error bars for each
datapoint
datapoints_dict, error_dict = abundance_through_time_by_feeding(groups_to_test,
week_dfs, all_samples_bifido_abun_df,
                                                                master_res_gene_metadata)

# Generate figure to show the abundance of Bifidobacterium over time
fig = plt.figure(figsize=(8,4))
ax1 = plt.subplot(2,1,1)
plt.title("Bifidobacterium")
formula_points = datapoints_dict['bifidobacterium'][0]
breast_points = datapoints_dict['bifidobacterium'][1]
ax1.errorbar(x=np.arange(0,5), y=formula_points, yerr=error_dict['bifidobacterium'][0],
color='red')
ax1.errorbar(x=np.arange(0,5), y=breast_points, yerr=error_dict['bifidobacterium'][1],
color='blue')
formula = mpatches.Patch(color='red', label='received formula only')
breast = mpatches.Patch(color='blue', label='received breastmilk')
leg = plt.legend(handles=[formula, breast], loc='upper left', prop={'size': 10})
leg.get_frame().set_linewidth(0.0)
plt.ylabel('Relative abundance')
plt.legend()
plt.ylim(-0.01,0.13)
plt.xlim(-0.2,4.2)
ax1.set_xticklabels(["",2,3,4,5,6])
ax1.yaxis.set_ticks(np.arange(0, 0.15, 0.03))
for item in ([ax1.title, ax1.xaxis.label, ax1.yaxis.label] +
             ax1.get_xticklabels() + ax1.get_yticklabels()):
    item.set_fontsize(10)
plt.show()
```

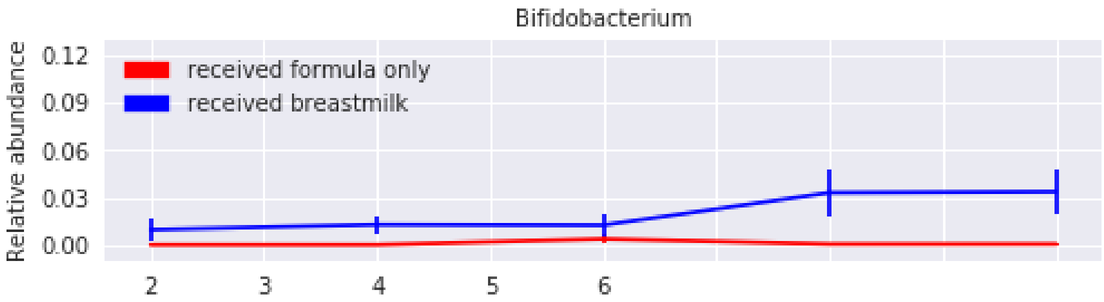

0.1.11 Exploring the relationship between replication rate and antibiotic resistance

The replication rate of a microbial population can be inferred by calculating an index of replication (iRep). This section elucidates the influence that antibiotic resistance genes may have on the replication rate of bacteria. First, we explore whether the total resistance content of a sample is correlated with the mean replication rate. Then, we test all resistance gene mechanisms to determine if genes of a particular category are intrinsicially associated with higher iRep for a genome, and repeat this analysis within individual phyla.

```
In [197]: def add_phylum(df):
              """For an input dataframe with a column containing the taxonomy, extract the phylum
```

```
    from the taxonomy string and add it as a new column in the dataframe."""
    split_taxonomy_strings = df['taxonomy_string'].str.split("|")
    phyla = []
    for entry in split_taxonomy_strings:
        try:
            phylum = entry[3]
            phyla.append(phylum)
        except IndexError:
            phylum = ""
            phyla.append(phylum)
    df['phylum'] = phyla
    return df

def after_abx_samples(df):
    """From a dataframe containing both sample resistance gene summaries and metadata,
return
    a dataframe of samples that were taken within five days after postweek antibiotic
treatment."""
    abx_babies_data = df.loc[df.postweek_ab == True]
    abx_babies_postweek_data = abx_babies_data.loc[abx_babies_data.DOL > 7]
    samples_with_abx = abx_babies_postweek_data.loc[abx_babies_postweek_data.antibiotics
!= '[]']
    infant_to_DOLs = {}
    for infant in samples_with_abx.infant:
        list_of_DOLs = []
        this_infant = samples_with_abx.loc[samples_with_abx.infant == infant]
        infant_to_DOLs[infant] = []
        for DOL in this_infant.DOL:
            infant_to_DOLs[infant].append(DOL)
    DOLs_after_abx = {}
    for infant in list(infant_to_DOLs.keys()):
        list_of_DOLs = infant_to_DOLs[infant]
        DOLs_after_abx[infant] = []
        for DOL in list_of_DOLs:
            DOL_plus1 = DOL + 1
            DOLs_after_abx[infant].append(DOL_plus1)
            DOL_plus2 = DOL + 2
            DOLs_after_abx[infant].append(DOL_plus2)
            DOL_plus3 = DOL + 3
            DOLs_after_abx[infant].append(DOL_plus3)
            DOL_plus4 = DOL + 4
            DOLs_after_abx[infant].append(DOL_plus4)
            DOL_plus5 = DOL + 5
            DOLs_after_abx[infant].append(DOL_plus5)
        DOLs_after_abx[infant] = list(set(DOLs_after_abx[infant])) #unique DOLs
    samples_5days_after = []
    for infant in list(DOLs_after_abx.keys()):
        this_infant = abx_babies_postweek_data.loc[abx_babies_postweek_data['infant'] ==
infant]
        after_abx_samples =
this_infant.loc[this_infant.DOL.isin(DOLs_after_abx[infant])]
        samples_5days_after.append(after_abx_samples)
    after_abx_df = pd.concat(samples_5days_after)
    return(after_abx_df)

def mean_sample_iRep(iRep_df, samples_of_interest_df):
    """Using a dataframe containing iRep values, find the mean iRep for each sample and
subset
    to only include samples listed in a samples of interest dataframe."""
    mean_iRep_for_samples = iRep_df.groupby('sample')['iRep'].mean()
    mean_iRep_for_samples = mean_iRep_for_samples.to_frame()
    mean_iRep_for_samples = mean_iRep_for_samples.reset_index(level=0)
    mean_iRep_for_samples_of_interest = mean_iRep_for_samples.loc \
[mean_iRep_for_samples['sample'].isin(samples_of_interest_df['sample'])]
    return(mean_iRep_for_samples_of_interest)

iRep_filter = iRep_info.loc[iRep_info['iRep'] < 3] # iRep values over 3 are unreliable
and may indicate genome
```

```
                                                    # contamination/strain variation
iRep_df = add_phylum(iRep_filter)
after_abx_res_gene_metadata = after_abx_samples(master_res_gene_metadata)
mean_iRep_for_after_abx_samples = mean_sample_iRep(iRep_df, after_abx_res_gene_metadata)
# Create scatterplot showing the relationship between sample's mean iRep and total
resistance gene content,
    # for samples within five days of postweek antibiotic treatment
sample_resistance_gene_summaries_fixed = sample_resistance_gene_summaries.drop('index',
axis=1)
sample_resistance_gene_summaries_fixed['total'] = \
sample_resistance_gene_summaries_fixed.drop('sample',axis=1).astype(float).sum(axis=1)
iRep_and_ARG =
mean_iRep_for_after_abx_samples.merge(sample_resistance_gene_summaries_fixed,
on='sample')
# withdraw samples influenced by over-representation of resistant bugs
iRep_and_ARG = iRep_and_ARG.loc[iRep_and_ARG['total'] < 1400]
plt.figure(figsize=(7,6))
sns.regplot(iRep_and_ARG['iRep'],iRep_and_ARG['total'], ci=0)
plt.ylabel('antibiotic resistance counts per million reads')
plt.xlabel('average replication rate in sample')
plt.show()

pearson_r, p_value = scipy.stats.pearsonr(iRep_and_ARG['iRep'], iRep_and_ARG['total'])
print("Pearson's r = ", pearson_r, "p = ", p_value)
```

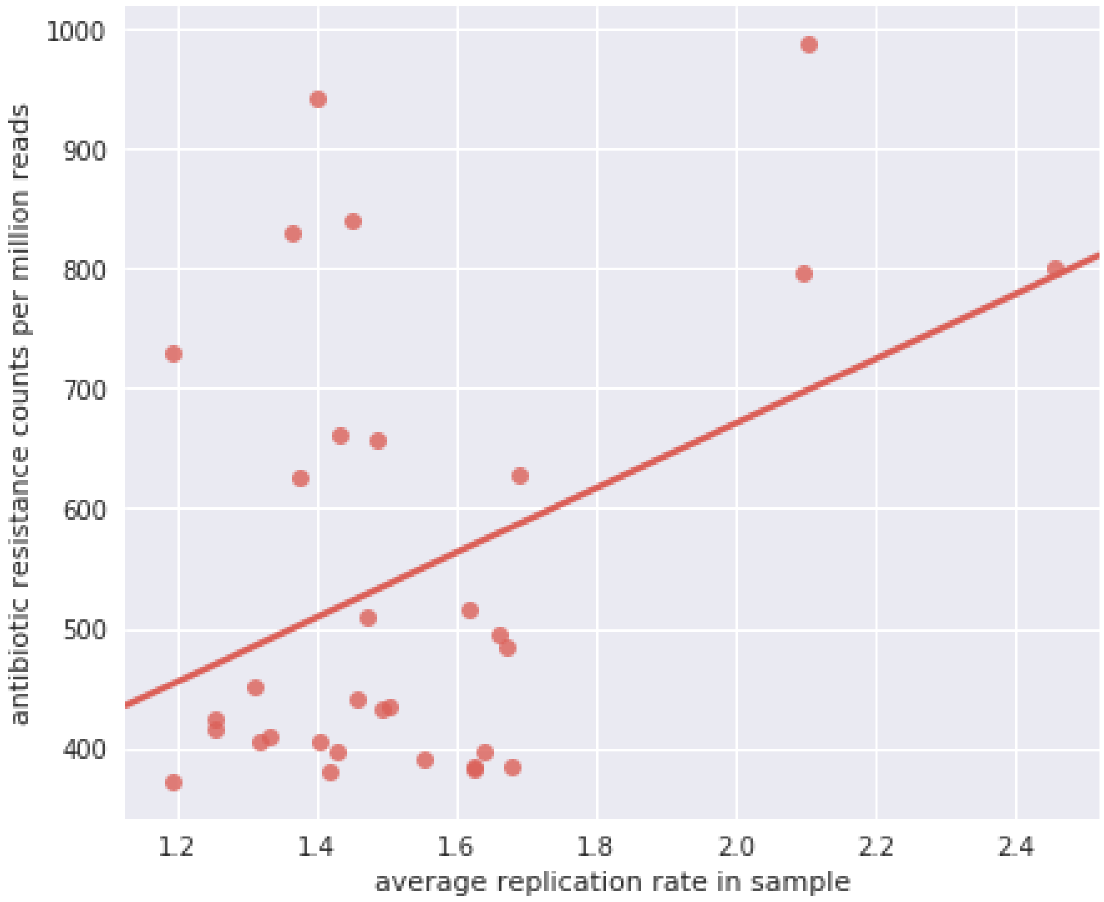


```
Pearson's r =  0.393594869605 p =  0.0258303132018
```

In [204]:
```
# Look only at samples of infants that did not receive postweek antibiotics to avoid
confounding effects
samples_of_no_abx_babies =
master_res_gene_metadata.loc[master_res_gene_metadata['postweek_ab'] == False]['sample']
iRep_df_of_no_abx_babies = iRep_df.loc[iRep_df['sample'].isin(samples_of_no_abx_babies)]
genome_res_by_category['genome'] = genome_res_by_category.index

def test_iRep_difference(iRep_df, genome_res_df):
    """Performs a Mann-Whitney U test on the iRep values of genomes harboring the
resistance gene/mechanism
    of interest and those that lack this gene/mechanism within the same phylum,
    followed by Bonferonni corrections to adjust for multiple testing."""
    res_to_result_dict = {} # Dictionary containing the results of Mann-Whitney U test
on iRep values of genomes
    p_values = []
    u_statistics = []
    for res_of_interest in genome_res_by_category.columns:
        if res_of_interest != 'genome':
            plus_genome =
genome_res_by_category.loc[genome_res_by_category[res_of_interest] > 0]['genome'] #has
gene
            minus_genome =
genome_res_by_category.loc[genome_res_by_category[res_of_interest] == 0]['genome']
#lacks it
            plus_iRep = iRep_df.loc[iRep_df['genome'].isin(plus_genome)]['iRep']
            minus_iRep = iRep_df.loc[iRep_df['genome'].isin(minus_genome)]['iRep']
            u_statistic, p_value = scipy.stats.mannwhitneyu(plus_iRep, minus_iRep)
            p_values.append(p_value)
            u_statistics.append(u_statistic)
    correct_ps = statsmodels.sandbox.stats.multicomp.multipletests(p_values,
method='bonferroni')[1]
    i = 0
    for res_of_interest in genome_res_by_category.columns:
        if res_of_interest != 'genome':
            res_to_result_dict[res_of_interest] = [u_statistics[i], correct_ps[i]]
            i += 1
    phylum_dict = {}
    p_values = []
    u_statistics = []
    num_comparisons = 0
    for key,value in res_to_result_dict.items():
        if value[1] < 0.05:
            # this code block counts the comparisons that need to be made
            # required for my implementation of Bonferonni correction, used rather than
built-in
            # statsmodels version due to dictionary-induced loss of order
            plus_genome = genome_res_by_category.loc[genome_res_by_category[key] >
0]['genome'] #has gene
            minus_genome = genome_res_by_category.loc[genome_res_by_category[key] ==
0]['genome'] #lacks gene
            plus_iRep_df = iRep_df.loc[iRep_df['genome'].isin(plus_genome)]
            minus_iRep_df = iRep_df.loc[iRep_df['genome'].isin(minus_genome)]
            phyla_in_plus = set(plus_iRep_df['phylum'])
            phyla_in_minus = set(minus_iRep_df['phylum'])
            phyla_of_both = phyla_in_plus.intersection(phyla_in_minus)
            num_comparisons += len(phyla_of_both)
    for key,value in res_to_result_dict.items():
        if value[1] < 0.05:
            # for significant category, find members of the same phylum that do and do
not have this gene
            # and perform mann-whitney u-test followed by Bonferonni correction
            plus_genome = genome_res_by_category.loc[genome_res_by_category[key] >
0]['genome'] #has gene
            minus_genome = genome_res_by_category.loc[genome_res_by_category[key] ==
0]['genome'] #lacks gene
            plus_iRep_df = iRep_df.loc[iRep_df['genome'].isin(plus_genome)]
            minus_iRep_df = iRep_df.loc[iRep_df['genome'].isin(minus_genome)]
            phyla_in_plus = set(plus_iRep_df['phylum'])
```

```
            phyla_in_minus = set(minus_iRep_df['phylum'])
            phyla_of_both = phyla_in_plus.intersection(phyla_in_minus)
            for phylum in phyla_of_both:
                iRep_plus = plus_iRep_df.loc[plus_iRep_df['phylum']==phylum]['iRep']
                iRep_minus = minus_iRep_df.loc[minus_iRep_df['phylum']==phylum]['iRep']
                u_statistic, p_value = scipy.stats.mannwhitneyu(iRep_plus, iRep_minus)
                corrected_p = bonferroni_correction(p_value, num_comparisons)
                phylum_dict[phylum] = [key, u_statistic, corrected_p]
    return(phylum_dict)

def setBoxColors(bp):
    setp(bp['boxes'][0], color='purple')
    setp(bp['caps'][0], color='purple')
    setp(bp['caps'][1], color='purple')
    setp(bp['whiskers'][0], color='purple')
    setp(bp['whiskers'][1], color='purple')
    setp(bp['medians'][0], color='purple')

    setp(bp['boxes'][1], color='orange')
    setp(bp['caps'][2], color='orange')
    setp(bp['caps'][3], color='orange')
    setp(bp['whiskers'][2], color='orange')
    setp(bp['whiskers'][3], color='orange')
    setp(bp['medians'][1], color='orange')

phylum_dict = test_iRep_difference(iRep_df_of_no_abx_babies, genome_res_by_category)
# information in phylum_dict indicates that bacteria harboring MFS are significantly
faster replicators than
# bacteria lacking this gene

# Create boxplot displaying the difference in iRep between bacteria with and without MFS
genes
MFS_plus_genome = genome_res_by_category.loc[genome_res_by_category['MFS
Transporter']>0].index
MFS_minus_genome = genome_res_by_category.loc[genome_res_by_category['MFS
Transporter']==0].index
MFS_plus_iRep_df =
iRep_df_of_no_abx_babies.loc[iRep_df_of_no_abx_babies['genome'].isin(MFS_plus_genome)]
MFS_plus_iRep = MFS_plus_iRep_df['iRep']
MFS_minus_iRep_df =
iRep_df_of_no_abx_babies.loc[iRep_df_of_no_abx_babies['genome'].isin(MFS_minus_genome)]
MFS_minus_iRep = MFS_minus_iRep_df['iRep']
MFS_minus_iRep_df_Firmicutes =
MFS_minus_iRep_df.loc[MFS_minus_iRep_df['phylum']=='Firmicutes']
MFS_plus_iRep_df_Firmicutes =
MFS_plus_iRep_df.loc[MFS_plus_iRep_df['phylum']=='Firmicutes']
data = [MFS_plus_iRep, MFS_minus_iRep]
u_1, p_1 = scipy.stats.mannwhitneyu(MFS_plus_iRep, MFS_minus_iRep)
p_1_corrected = bonferroni_correction(p_1, 13)
print('In all Bacteria, members with MFS replicate faster \
      \n (Mann Whitney U = '+str(u_1)+', p = '+str(p_1_corrected)+')')
data2 = [MFS_plus_iRep_df_Firmicutes['iRep'], MFS_minus_iRep_df_Firmicutes['iRep']]
u_2, p_2 = scipy.stats.mannwhitneyu(MFS_plus_iRep_df_Firmicutes['iRep'],
MFS_minus_iRep_df_Firmicutes['iRep'])
p_2_corrected = bonferroni_correction(p_2, 20)
print('Within the Firmicutes phylum, members with MFS replicate faster \
      \n (Mann Whitney U = '+str(u_2)+', p = '+str(p_2_corrected)+')')

fig = plt.figure(figsize=(6,5))
ax = plt.axes()
# first boxplot pair
bp = plt.boxplot(data, positions = [1, 2], widths = 0.6, showfliers=False,
patch_artist=True)
setBoxColors(bp)
for median in bp['medians']:
    median.set(color='black', linewidth=0.8,)
# second boxplot pair
bp = plt.boxplot(data2, positions = [4, 5], widths = 0.6, showfliers=False,
```

```
patch_artist=True)
setBoxColors(bp)
# set axes limits and labels
xlim(0,6)
ylim(1,2.6)
ax.set_xticklabels(['all Bacteria', 'Firmicutes'])
ax.set_xticks([1.5, 4.5])
hB, = plot([1,1],'purple')
hR, = plot([1,1],'orange')
plt.legend((hB, hR),('harbors MFS', 'lacks MFS'),loc='upper left')
hB.set_visible(False)
hR.set_visible(False)
leg = plt.gca().get_legend()
llines = leg.get_lines()
for median in bp['medians']:
    median.set(color='black', linewidth=0.8,)
plt.setp(llines, linewidth=5.5)
plt.gca().xaxis.grid(False)
plt.ylabel('replication rate (iRep)', labelpad=12)
plt.show()
```

```
In all Bacteria, members with MFS replicate faster
 (Mann Whitney U = 827176.0, p = 1.55076289871e-05)
Within the Firmicutes phylum, members with MFS replicate faster
 (Mann Whitney U = 136756.0, p = 0.000217531513864)
```

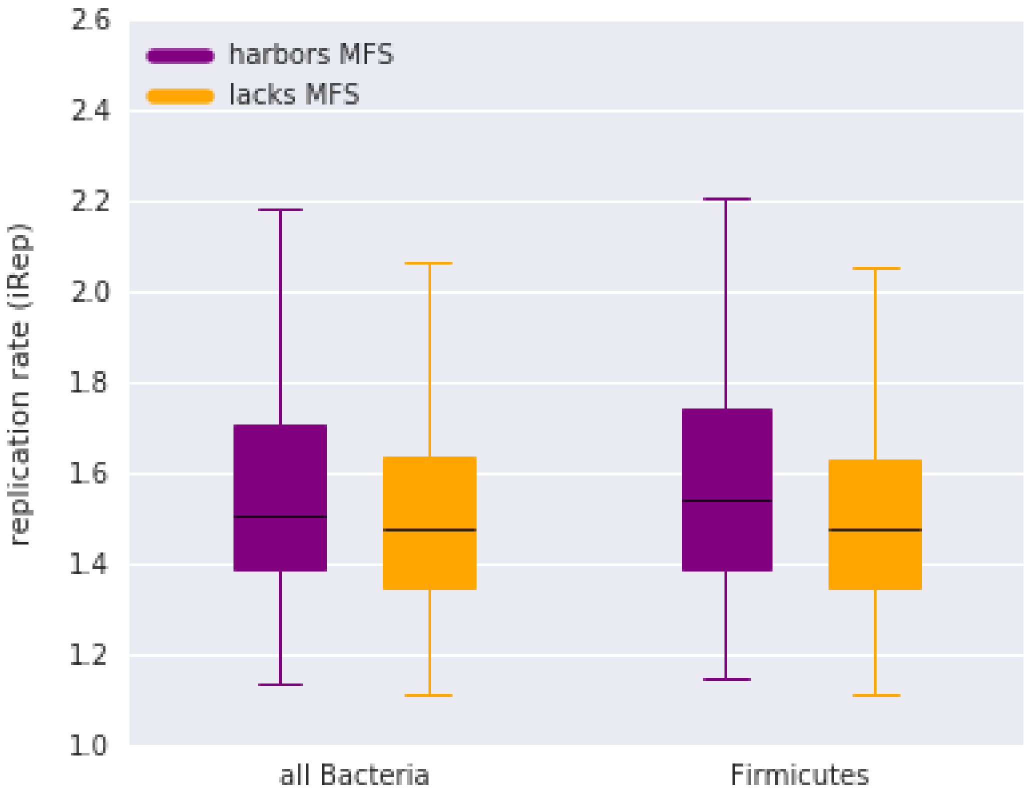

In [205]:
```
# Check if there is a significant different in the completeness (measured by presence of
# bacterial single copy genes) of the genomes with and without MFS
```

```
MFS_plus_genome_metrics = genome_metrics.loc[genome_metrics['Bin
Id'].isin(MFS_plus_genome)]
MFS_minus_genome_metrics = genome_metrics.loc[genome_metrics['Bin
Id'].isin(MFS_minus_genome)]
U_stat, p_val = scipy.stats.mannwhitneyu(MFS_plus_genome_metrics['Completeness'],
MFS_minus_genome_metrics['Completeness'])
print("There is a significant difference in the completeness of the two groups, p
=",p_val)
if MFS_plus_genome_metrics['Completeness'].mean() >
MFS_minus_genome_metrics['Completeness'].mean():
    winner ='Genomes that have MFS'
else:
    winner = 'Genomes lacking MFS'
print(winner,"have higher completeness.")
```

```
There is a significant difference in the completeness of the two groups, p =
1.28116449442e-12
Genomes lacking MFS have higher completeness.
```

0.1.12 Predicting a gut organism's response to antibiotics

This is a model that uses the gene content of a gut organism to predict its direction of change in relative abundance (increase vs. decrease) after a premature infant is administered a combination of vancomycin and cephalosporin antibiotics. To land upon this model, several rounds of adjusting model scope, engineering, transforming, & selecting features, varying algorithm choice, and tuning parameters were employed. However, only the final model (AdaBoost algorithm applied to five pricipal components of combined Resfams and KEGG data) is shown here.

```
In [207]: def label_class(list_of_dfs, infant_to_info_dict):
              """Based on a genome's abundances before antibiotics and after antibiotics,
              return a dictionary that indicates whether it increased or decreased in
          abundance."""
              def make_genome2samples_dict(list_of_dfs, infant_to_info_dict):
                  """Intake a list of dataframes containing time-series relative abundance data
          for each infant,
                  and return a dictionary with genomes as keys and the pre- & post- antibiotic
          samples in a list
                  as the dictionary's values."""
                  def identify_genomes(df):
                      """Select the genomes to be used in the model."""
                      for sample in df.columns:
                          for before_sample in before_samples:
                              if sample.startswith(before_sample):
                                  before_abx_genome_abundances = df[sample]
                                  selected_genomes = []
                                  for number in before_abx_genome_abundances:
                                      if number >= 0.0001: # include genome if it was detectable
          above this
                                                           # relative abundance level in the pre-
          antibiotic sample
                                          genome = df.loc[df[sample]==number]['genome']
                                          genome = genome.to_string()
                                          genome = genome.split(" ")[-1]
                                          genome_before_after[genome] = [number]
                                          selected_genomes.append(genome)
                                  for genome in selected_genomes:
                                      genome_of_interest_abundances = df.loc[df['genome']==genome]
                                      for after_sample in after_samples:
                                          for sample in genome_of_interest_abundances.columns:
                                              if sample.startswith(after_sample):
                                                  genome_abun_after_abx =
          float(genome_of_interest_abundances[sample])
```

```
genome_before_after[genome].append(genome_abun_after_abx)
        # create lists of the samples before antibiotics and the samples after
antibiotics
        before_samples = []
        after_samples = []
        for infant in infant_to_info:
            before = infant_to_info[infant][1]
            before_samples.append(before)
            after = infant_to_info[infant][2]
            after_samples.append(after)
        infants_of_interest = infant_to_info.keys() # these are the infants from which
samples will be extracted
        genome_before_after = {}
        for rel_abun_df in list_of_dfs:
            identify_genomes(rel_abun_df)
        return(genome_before_after) # dictionary with genome (key) and relative
abundance information in values
    genome_before_after = make_genome2samples_dict(list_of_dfs, infant_to_info_dict)
    genome_to_change = {}
    for genome in genome_before_after:
        difference = genome_before_after[genome][1] - genome_before_after[genome][0]
        # negative difference indicates decrease, positive difference indicates increase
        if difference > 0:
            change = 'increase'
        elif difference < 0:
            change = 'decrease'
        genome_to_change[genome+'.fa'] = change
    return(genome_to_change)

def merge_resfams_and_kegg(class_label_dict, input_Resfam, input_KEGG):
    """Combine the dataframes of the two types of annotations used (Resfams for
resistance genes and
    KEGG for general metabolism) and return one merged dataframe."""
    KEGG_results_df_t = input_KEGG.transpose()
    KEGG_results_df_t.columns = KEGG_results_df_t.ix[0]
    KEGG_results_df_t['genome'] = KEGG_results_df_t.index
    merged_df = KEGG_results_df_t.merge(input_Resfam, on='genome') #combine Resfams
annotations with KEGG annotations
    genome_to_change_df = pd.DataFrame.from_dict(class_label_dict,orient='index')
    genome_to_change_df['genome'] = genome_to_change_df.index
    merged_change_res_df = merged_df.merge(genome_to_change_df,on='genome') #add class
labels (increase vs decrease)
    merged_change_res_df = merged_change_res_df.drop('genome', axis=1)
    return(merged_change_res_df)

def input_and_output(class_label_dict, input_Resfam, input_KEGG):
    """Using a dictionary containing class labels, return an array containing the
    Resfams/KEGG annotations for each genome (gene_array) and a corresponding array
    containing the numerically encoded class label for each genome (Y)"""
    merged_change_res_df = merge_resfams_and_kegg(class_label_dict, input_Resfam,
input_KEGG)
    Y = merged_change_res_df[0] # output class labels
    gene_df = merged_change_res_df.drop(0, axis=1)
    gene_array = gene_df.values # input/predictor variables
    le = sklearn.preprocessing.LabelEncoder()
    le.fit(Y)
    Y = le.transform(Y) # convert "increase" to 1 and "decrease" to 0
    return(gene_array, Y)

infant_to_info = {} # A dictionary with infants and keys and values including 1) the
antibiotic combination,
                     # 2) the name of the sample before antibiotics, and 3) the name of
the post-antibiotic sample
# Six infants in our dataset were administered an antibiotic combination of vancomycin
and cephalosporins
infant_to_info['N1_003'] = [['vancomycin', 'cefazolin'], 'N1_003_044G1', 'N1_003_052G1']
infant_to_info['N1_019'] = [['vancomycin', 'claforan'], 'N1_019_022G1', 'N1_019_024G1']
infant_to_info['N4_097'] = [['vancomycin', 'claforan'], 'N4_097_010G1', 'N4_097_027G1']
```

```
infant_to_info['N4_101'] = [['vancomycin', 'claforan'], 'N4_101_015G1', 'N4_101_017G1']
infant_to_info['N1_008'] = [['vancomycin', 'cefazolin'], 'N1_008_008G1', 'N1_008_010G1']
infant_to_info['N1_011'] = [['vancomycin', 'cefazolin'], 'N1_011_033G1', 'N1_011_037G1']
N1_003_rel_abun = pd.read_csv("relative_abundance_csvs/N1_003_rel_abun.csv")
N1_008_rel_abun = pd.read_csv("relative_abundance_csvs/N1_008_rel_abun.csv")
N1_019_rel_abun = pd.read_csv("relative_abundance_csvs/N1_019_rel_abun.csv")
N4_101_rel_abun = pd.read_csv("relative_abundance_csvs/N4_101_rel_abun.csv")
N4_097_rel_abun = pd.read_csv("relative_abundance_csvs/N4_097_rel_abun.csv")
N1_011_rel_abun = pd.read_csv("relative_abundance_csvs/N1_011_rel_abun.csv")
list_rel_abun_dfs = [N1_003_rel_abun, N1_008_rel_abun, N1_019_rel_abun,
                     N4_101_rel_abun, N4_097_rel_abun, N1_011_rel_abun]

genome_before_after = label_class(list_rel_abun_dfs, infant_to_info)
genome_resistance_gene_summaries['genome'] = genome_resistance_gene_summaries.index
X_gene_array, Y = input_and_output(genome_before_after,
genome_resistance_gene_summaries, KEGG_genome_profiles)

# Split into train and test final
X_train, X_test_final, y_train, y_test_final = \
sklearn.model_selection.train_test_split(X_gene_array, Y, test_size=0.15, stratify=Y,
random_state=123)

# Split training data into train_final and test_validation
X_train_final, X_test_validation, y_train_final, y_test_validation = \
sklearn.model_selection.train_test_split(X_train, y_train, test_size=0.15,
stratify=y_train, random_state=123)

# Transform input arrays into five principal components
pca = PCA(n_components=5, random_state=123)
# Fit/transform each set separately to avoid training classifier with data based on a
fit that includes test sets
X_test_validation_PCs = pca.fit(X_test_validation).transform(X_test_validation)
X_test_final_PCs = pca.fit(X_test_final).transform(X_test_final)
X_train_final_PCs = pca.fit(X_train_final).transform(X_train_final)

# Create plot to show the % variation explained by the principal components
plt.figure(figsize=(4, 3))
plt.plot(np.cumsum(pca.explained_variance_), linewidth=2)
plt.axis('tight')
plt.xticks([0, 1, 2, 3, 4],[1, 2, 3, 4, 5])
plt.ylim(0,100)
plt.xlabel('number of principal components')
plt.ylabel('cumulative explained variance')
plt.show()

print('Principal components generated from combined Resfams (resistance) and KEGG
(overall metabolism) annotations.')
print('Five principal components captures ~48% of the variation')
```

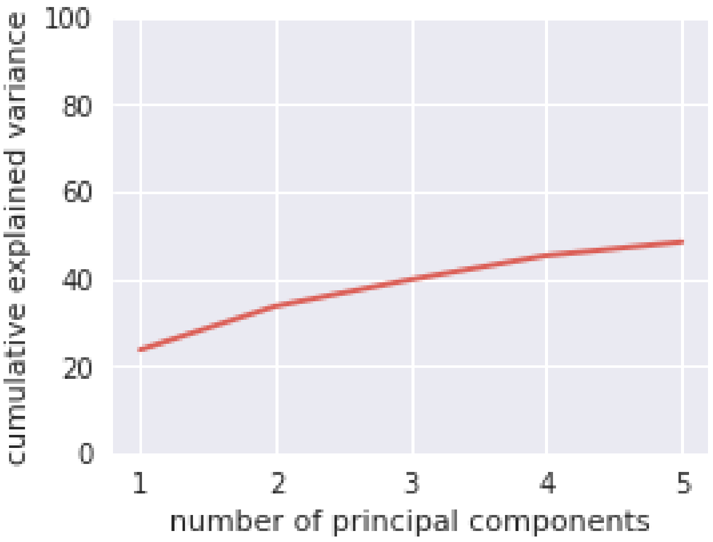


```
Principal components generated from combined Resfams (resistance) and KEGG (overall
metabolism) annotations.
Five principal components captures ~48% of the variation
```

```
In [208]: # This classifier and parameters were selected after multiple rounds of experimentation
          and tests on the validation set
          classifier = AdaBoostClassifier(algorithm='SAMME.R',
                   learning_rate=1.0, n_estimators=50, random_state=123)

          # Testing on validation set
          classifier.fit(X_train_final_PCs,y_train_final) # train model
          predictions = classifier.predict(X_test_validation_PCs) # make predictions
          precision, recall, fscore, support = score(y_test_validation, predictions) # evaluate
          predictions
          precision = precision.mean()
          recall = recall.mean()
          print('Validation set results: \n (note: validation set was used in prior instances of
          model testing and refinement, \
                \n but was not used to train the model)')
          print('precision: {0:.1f}'.format(precision))
          print('recall: {0:.1f}\n'.format(recall))

          # Testing on final test set
          classifier.fit(X_train_final_PCs,y_train_final) # train model
          predictions = classifier.predict(X_test_final_PCs) # make predictions
          precision, recall, fscore, support = score(y_test_final, predictions) # evaluate
          predictions
          precision = precision.mean()
          recall = recall.mean()
          print('Test set results (completely unseen data):')
          print('precision: {0:.1f}'.format(precision))
          print('recall: {0:.1f}'.format(recall))
```

```
Validation set results:
 (note: validation set was used in prior instances of model testing and refinement,
 but was not used to train the model)
precision: 1.0
recall: 1.0
```

```
Test set results (completely unseen data):
precision: 0.9
recall: 0.7
```

In [209]:
```
# Determine which genes were the strongest contributors to the principal components used
as input to the model
# and were indicative of a genome's tendency to increase after antibiotic administration

def identify_PC_contributors(pca_components, merged_change_res_df):
    """Return the names of the genes or modules that are within the top five
contributors of
    at least one of the principal components used as model input."""
    merged_gene_df = merged_change_res_df.drop(0, axis=1)
    PC_RF_corr = pd.DataFrame(pca_components, columns=merged_gene_df.columns) # convert
PCs to dataframe
    PC_RF_corr_abs = PC_RF_corr.abs() # convert to absolute value to determine PC
strongest contributors
    PC_to_top_five = {} # dictionary containing each PC (key) to its top five components
(values)
    PC_num = 1
    for x in range(0,5):
        selected_PC = PC_RF_corr_abs.ix[x:x,:].sort_values(by=x,axis=1)
        top_five = selected_PC.ix[:,-5:]
        PC_to_top_five[PC_num] = top_five
        PC_num += 1
    five_pc_df = pd.concat(PC_to_top_five.values()) # generate dataframe from the
component dictionary
    return(five_pc_df.columns) # return the names of the strongest contributors

def calculate_class_tendency(merged_change_res_df, accessions_of_interest):
    """Of the genes that are the strongest contributors to the principal components,
    return the genes that are associated with the 'increase' class of genomes and return
    a value that represents their tendency to occur in this class"""
    increase_df = merged_change_res_df.loc[merged_change_res_df[0]=='increase']
    decrease_df = merged_change_res_df.loc[merged_change_res_df[0]=='decrease']
    accession_to_group = {}
    accession_to_ratio = {}
    accessions = []
    ratios = []
    # label whether a particular gene or module more frequently appeared in the
'increase' or 'decrease' group
    for accession in accessions_of_interest:
        if increase_df[accession].mean() > decrease_df[accession].mean():
            group = 'increase'
        else:
            group = 'decrease'
        accession_to_group[accession] = group
        ratio = increase_df[accession].mean()/decrease_df[accession].mean() #calculate
ratio of means
        accession_to_ratio[accession] = ratio
        accessions.append(accession)
        ratios.append(ratio)
    return(np.asarray(ratios))

def autolabel(rects, list_of_labels):
    """Give labels to bars of interest in bar graph."""
    labels = accessions_of_interest
    i = 0
    for rect in rects:
        if i == 11:
            height = rect.get_height()
            plt.text(rect.get_x() + 1.3, 1.05*height,
            list_of_labels[i], ha='center', va='bottom')
        elif i == 17:
            height = rect.get_height()
            plt.text(rect.get_x() - 0.5, 1.05*height,
```

```
            list_of_labels[i], ha='center', va='bottom')
        elif i == 18:
            height = rect.get_height()
            plt.text(rect.get_x() + 0.7, 1.05*height,
            list_of_labels[i], ha='center', va='bottom')
        else:
            height = rect.get_height()
            plt.text(rect.get_x() + rect.get_width()/2., 1.05*height,
                    list_of_labels[i], ha='center', va='bottom')
        i = i+1

merged_change_res_df = merge_resfams_and_kegg(genome_before_after,
genome_resistance_gene_summaries, \
                                               KEGG_genome_profiles)
accessions_of_interest = identify_PC_contributors(pca.components_, merged_change_res_df)
class_tendency_data = calculate_class_tendency(merged_change_res_df,
accessions_of_interest)

#Plot results
genes_for_bars = []
for accession in accessions_of_interest:
    genes_for_bars.append(accession_to_gene_dict[accession])
l_labels = list(" "*len(genes_for_bars)) # initialize list of empty strings
# label the bars that correspond to the increase class (names  from 'genes for bars'
were abbreviated manually)
l_labels[7] = "ABC efflux"
l_labels[10] = 'mexH'
l_labels[11] = 'mexW/mexI'
l_labels[17] = 'B2 B-L'
l_labels[18] = 'VanR'
baseline = 1
mask = class_tendency_data <= 1
colors = np.array(['r']*len(class_tendency_data))
colors[mask] = 'k'
ind = range(len(class_tendency_data))
plt.figure(figsize=(6,4))
bg = plt.bar(ind,[x - baseline for x in class_tendency_data], width=0.2, color=colors)
plt.xlim(-0.5,20)
plt.ylim(-0.7,0.9)
plt.ylabel('class tendency')
plt.gca().xaxis.grid(False)
plt.xticks([])
autolabel(bg, l_labels)
plt.rcParams.update({'font.size': 9})
plt.show()
```

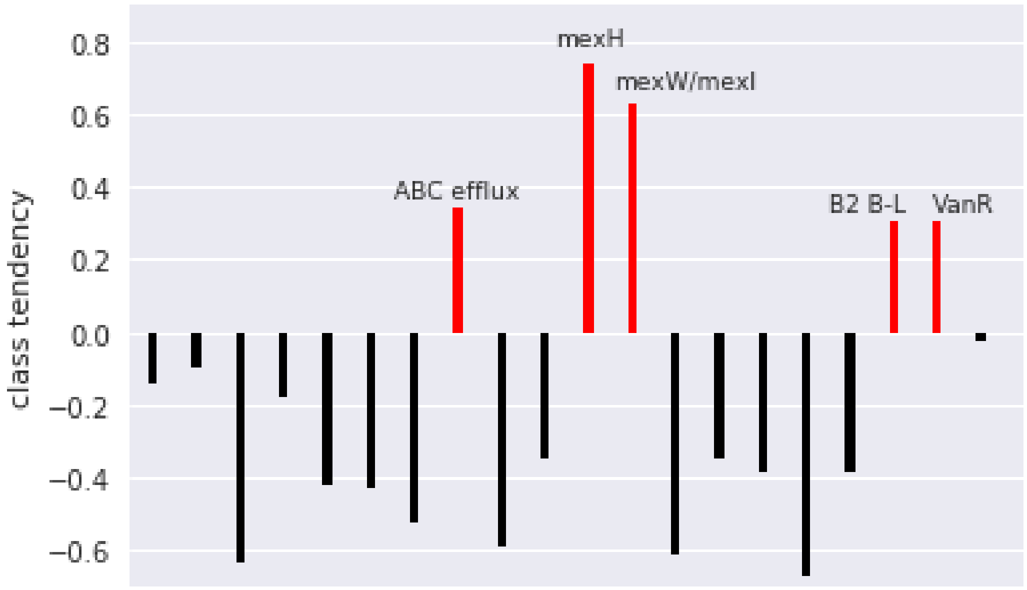


```
In [210]: # Which antibiotic-resistant organisms harbored the genes that indicated tendency to
          increase in abundance

          # First, identify all the mircobes that were in the increase class
          genomes_that_increase = []
          for genome, result in genome_before_after.items():
              if result == 'increase':
                  genomes_that_increase.append(genome)
          #of these genomes, match them with res gene and taxonomy, and identify the ones that
          carried genes of interest
          genome_res_tax_increase_df = genome_resistance_gene_summaries_tax.loc \
          [genome_resistance_gene_summaries_tax['genome'].isin(genomes_that_increase)]
          for i, accession in enumerate(accessions_of_interest):
              if class_tendency_data[i] > 1: # if this accession is associated with the increase
          group
                  print(str(accession_to_gene_dict[accession]))
                  #determine which genomes had the highest counts of this gene
          print(list(genome_res_tax_increase_df.sort_values(by=accession)[-3:]['taxonomy']),"\n")
```

```
    ATP-binding cassette (ABC) antibiotic efflux pump
['Bradyrhizobium sp. BTAi1', 'Enterococcus faecalis', 'Clostridium baratii str.
Sullivan']

08    mexH: membrane fusion protein of the efflux co...
['Bacteroides ovatus', 'Bacteroides helcogenes P 36-108', 'Bradyrhizobium sp. BTAi1']

09    A grouping of related mexW and mexI subunits o...
['Bacteroides ovatus', 'Bacteroides helcogenes P 36-108', 'Bradyrhizobium sp. BTAi1']

35    Subclass B2 (metallo-) beta-lactamase selectiv...
['Enterococcus faecalis V583', 'Clostridium baratii str. Sullivan', 'Bradyrhizobium
sp. BTAi1']

60    VanR: transcriptional activator regulating Van...
['Enterococcus faecalis', 'Clostridium perfringens', 'Clostridium baratii str.
Sullivan']
```
