# Supplementary material for: Machine Learning Leveraging Genomes from Metagenomes Identifies Influential Antibiotic Resistance Genes in the Infant Gut Microbiome
Source: mSystems. 2018 Jan 9;3(1):e00123-17. doi: 10.1128/mSystems.00123-17 (PMC5758725; doi:10.1128/mSystems.00123-17)
Supplement: TABLE S6 [file sys001182161st6.docx]

| **WEEK 2 (Table S6-A)** | **Degrees of freedom** | **Sum of Squares** | **F-statistic** | **p value** | **corrected p value** |
| --- | --- | --- | --- | --- | --- |
| received_breastmilk | 1 | 0.3163 | 2.2328 | 0.0506 | 0.1518 |
| infant_antibiotics | 1 | 0.3958 | 2.7941 | 0.023 | 0.069 |
| birth_mode | 1 | 0.1875 | 1.3237 | 0.2146 | 0.6438 |
| gender | 1 | 0.0968 | 0.6837 | 0.6444 | 1 |
| maternal_antibiotics | 1 | 0.2846 | 2.0089 | 0.0728 | 0.2184 |
| Residual | 83 | 11.7574 |  |  |  |

| **WEEK 4 (Table S6-B)** | **Degrees of freedom** | **Sum of Squares** | **F-statistic** | **p value** | **corrected p value** |
| --- | --- | --- | --- | --- | --- |
| **received_breastmilk** | **1** | **0.5348** | **4.2009** | **0.0026** | **0.0078** |
| infant_antibiotics | 1 | 0.0824 | 0.647 | 0.6049 | 1 |
| birth_mode | 1 | 0.2427 | 1.9069 | 0.0791 | 0.2373 |
| Gender | 1 | 0.1169 | 0.9182 | 0.4462 | 1 |
| maternal_antibiotics | 1 | 0.2132 | 1.6747 | 0.1152 | 0.3456 |
| Residual | 64 | 8.1471 |  |  |  |

| **WEEK 6 (Table S6-C)** | **Degrees of freedom** | **Sum of Squares** | **F-statistic** | **p value** | **corrected p value** |
| --- | --- | --- | --- | --- | --- |
| **received_breastmilk** | **1** | **0.4619** | **5.005** | **0.0024** | **0.0072** |
| infant_antibiotics | 1 | 0.0994 | 1.0765 | 0.3168 | 0.9504 |
| birth_mode | 1 | 0.0869 | 0.9411 | 0.4227 | 1 |
| gender | 1 | 0.1601 | 1.7351 | 0.1285 | 0.3855 |
| maternal_antibiotics | 1 | 0.05 | 0.5417 | 0.7404 | 1 |
| Residual | 25 | 2.307 |  |  |  |
